# Hybrid Disease Diagnosis Using Multiobjective Optimization with Evolutionary Parameter Optimization

**DOI:** 10.1155/2017/5907264

**Published:** 2017-07-04

**Authors:** MadhuSudana Rao Nalluri, Kannan K., Manisha M., Diptendu Sinha Roy

**Affiliations:** ^1^SASTRA University, Thanjavur, Tamil Nadu, India; ^2^National Institute of Technology, Meghalaya, India

## Abstract

With the widespread adoption of e-Healthcare and telemedicine applications, accurate, intelligent disease diagnosis systems have been profoundly coveted. In recent years, numerous individual machine learning-based classifiers have been proposed and tested, and the fact that a single classifier cannot effectively classify and diagnose all diseases has been almost accorded with. This has seen a number of recent research attempts to arrive at a consensus using ensemble classification techniques. In this paper, a hybrid system is proposed to diagnose ailments using optimizing individual classifier parameters for two classifier techniques, namely, support vector machine (SVM) and multilayer perceptron (MLP) technique. We employ three recent evolutionary algorithms to optimize the parameters of the classifiers above, leading to six alternative hybrid disease diagnosis systems, also referred to as hybrid intelligent systems (HISs). Multiple objectives, namely, prediction accuracy, sensitivity, and specificity, have been considered to assess the efficacy of the proposed hybrid systems with existing ones. The proposed model is evaluated on 11 benchmark datasets, and the obtained results demonstrate that our proposed hybrid diagnosis systems perform better in terms of disease prediction accuracy, sensitivity, and specificity. Pertinent statistical tests were carried out to substantiate the efficacy of the obtained results.

## 1. Introduction

The proliferations of computer usage across all aspects of life have resulted in accumulating a large number of systematic and related data. This has necessitated identifying useful patterns from raw datasets as the next logical step forward. Thus, data mining, a broad discipline encompassing classification, clustering, association, prediction, estimation, and visualization tasks [1], has emerged as a dynamic and significant field of research to address theoretical challenges as well as practical issues. Data mining and knowledge engineering techniques have been successfully applied to numerous areas, like education, pattern recognition, fraud detection, and medicine [2, 3].

The application of data mining and knowledge engineering techniques in the medical domain plays a prime role in the diagnosis of diseases and prognostication [4]. It assists healthcare professionals and doctors to analyze and predict diseases [5] and is often commonly referred to as medical engineering. Numerous machine learning algorithms have been developed to extract useful patterns from raw medical data over the years [6]. These patterns have been utilized for disease prediction using classification and clustering strategies. Medical research focuses on employing data mining for prediction of a broad range of diseases, including breast cancer [7], heart diseases [8], Parkinson's disease [9], hepatitis, and diabetes, only to name a few.

Over the years, several supervised machine learning techniques such as classification as well as several unsupervised machine learning techniques like clustering have been applied to available medical information [10, 11]. Individual classifiers, ensembles thereof, and hybrid systems have often been used to diagnose various diseases. Several techniques have been applied on medical data to improve such diagnosing efficacy, regarding performance parameters such as prediction accuracy, sensitivity, and specificity [12, 13].

This paper presents a hybrid system for diagnosis and prediction of numerous diseases using optimized parameters for classifiers. The classifier parameters are optimized using evolutionary algorithms to enhance classification performance. By juxtaposing the proposed parameter optimization step within existing classifier mechanisms, our method provides improved prediction accuracy. In this paper, 16 classifiers are executed in which two basics are with and without resampling, 6 hybrid intelligent systems without resampling and 6 hybrid intelligent systems with resampling technique. In summary, this paper presents a comparative analysis of parameter optimized versions of two classifiers, namely, support vector machine (SVM) and multilayer perceptron (MLP) for medical data. It has been concluded from experimental results presented in this paper that our proposed hybrid system outperforms state of the art (single or ensemble) for classifying medical data. To contrive the parameter optimization, we have employed three popular evolutionary algorithms, namely, particle swarm optimization (PSO), gravitational search algorithm (GSA), and firefly algorithm (FA) for optimizing parameters of SVM and MLP classifiers. Accordingly, we study the performance of six alternative hybrid systems for classifying medical data towards a diagnosis of such diseases. The performance of the proposed hybrid intelligent techniques is compared with the recent literature results (both simple and ensemble classifiers [14–16]). This hybrid intelligent system shows better performance than the recently published ensemble classifiers on 11 benchmark datasets.

The rest of this paper is organized as follows: A brief exposition of existing researches has been dealt with in Section 2, specifically focusing on several machine learning algorithms employed for processing medical datasets. The problem formulation of our proposed weighted multiobjective optimization for the classifying problem dealt with has been presented in Section 3.  Section 4 provides the rudimentary steps and key features of the evolutionary algorithms employed for the parameter optimization of SVM and MLP classifiers, namely, particle swarm optimization (PSO), gravitational search algorithm (GSA), and firefly algorithm (FA). A very basic introduction of the two classifiers employed, namely, SVM and MLP, has been discussed in Section 5.  Section 6 elaborately explains the development of the proposed hybrid classification system for disease diagnosis along with their key components and design principles involved. The performance of the proposed hybrid scheme is tested over 11 benchmark medical datasets, and Section 7 provides a brief account of the experimental setup and the experiments conducted and summarizes the results obtained. This section also presents a statistical analysis of obtained results for validating the acceptability of obtained statistical results. The conclusions of the research have been presented in Section 8.

## 2. Related Work

There have been abundant attempts to analyze and diagnose ailments employing machine learning algorithms. This section gives a summary of the efforts in this field to put the contribution of our work in perspective. These researches, however, vary considerably in terms of classifiers applied and nature of systems employed; for example, some are simple and others are hybrid whereas some others present ensemble systems. There are also major varieties in terms of objective functions chosen, single or multiobjective formulation, the number of datasets on which these methods have been applied, performance parameters employed for validating the efficacy, and so forth.

Among the different disease datasets that have been studied in the literature, heart disease diagnosis has been very prominent within medical engineering circles, and a wide variety of machine learning techniques have been explored towards diagnosing the same. References [17–38] include some prominent contributions towards diagnosing heart diseases from various aspects using myriad machine learning techniques, details of which are presented hereafter. Chitra and Seenivasagam [18] proposed a cascaded neural network (CNN) classifier and support vector machine (SVM) to diagnose heart diseases. The performance of CNN and SVM was compared based on the accuracy, sensitivity, and specificity. Pattekari and Parveen [19] suggested an intelligent system, which used a naive Bayes classifier that was further improved by developing ensemble-based classifiers. Das et al. [17] developed a neural network ensemble model for heart disease diagnosis. The proposed technique used Statistical Analysis System (SAS) enterprise guide 4.3 programs for data preprocessing and SAS Enterprise miner 5.2 programs for recognizing the heart disease by combining three neural networks ensemble. The technique was further improved by combining other neural networks and was also used for various datasets. Das et al. [37] described an SAS-based Software 9.1.3 for diagnosing valvular heart diseases. The proposed method used a neural network ensemble. Predicted values, posterior probabilities, and voting posterior probabilities were applied.

Masethe and Masethe [21] used J48, naive Bayes, REPTREE, CART, and Bayes Net for diagnosing the efficacy of heart diseases. High accuracy was obtained using a J48 tree. Shaikh et al. [22] evaluated the performance of three classifiers, namely, k-NN, naive Bayesian, and decision tree based on four parameters, namely, precision, recall, accuracy, and *F*-measure. k-NN produced higher accuracy than other methods. Bhatla and Jyoti [26] compared naive Bayes, decision tree, and neural networks for the said diagnosis. For the decision tree, genetic algorithm and fuzzy logic were employed, and results presented used TANAGRA tool.

Kavitha and Christopher [23] performed classification of heart rate using a hybrid particle swarm optimization and fuzzy C-means (PSO-FCM) clustering. The proposed method performed feature selection using PSO. The fuzzy C-means cluster and classifier are combined to enhance the accuracy. Enhanced SVM was used for classifying heart diseases. The hybrid system could be trained to shorten the implementation time. Alizadehsani et al. [24] evaluated sequential minimal optimization (SMO), naive Bayes, bagging with SMO, and neural networks. They employed rapid miner tool, and high accuracy was obtained using bagging with SMO. Abhishek [38] employed j48, naive Bayes, neural networks with all attributes for diagnosing heart diseases with the WEKA machine learning software and concluded that j48 outperformed others regarding accuracy.

Jabbar et al. [20] used association mining and genetic algorithm in conjunction with heart disease prediction. The proposed method used Gini index statistics for association algorithm and crossover, the mutation for the genetic algorithm. They further employed a feature selection technique for improved accuracy. Ordonez et al. [36] presented an improved algorithm to determine constrained association rules by two techniques: mapping medical data and identifying constraints. The proposed method used mining attributes. Constrained association rules and parameters were used for the mapping. The technique produced interesting results by comparing this association rule with classification rule. Shenfield and Rostami [25] introduced a multiobjective approach to the evolutionary design of artificial neural networks for predicting heart disease.

Parthiban and Subramanian [27] developed a coactive neurofuzzy inference system (CANFIS) for prediction of heart diseases. The proposed model combined CANFIS, neural network, and fuzzy logic. It was then integrated with a genetic algorithm. Results showed that GA was useful for autotuning of the CANFIS parameters. Hedeshi and Abadeh [28] performed PSO algorithm with a boosting approach. The proposed method used fuzzy rule extraction with PSO and enhanced-particle swarm optimization 2 (En-PSO2). Karaolis et al. [35] used myocardial infarction (MI), percutaneous coronary intervention (PCI), and coronary artery bypass graft surgery (CABG) models. The proposed method used C4.5 decision tree algorithms. Results were compared based on false positive (FP), precision, and so forth. By further investigation with various datasets and employing extraction rule algorithms further, better results were obtained.

Kim et al. [30] proposed a fuzzy rule-based adaptive coronary heart disease prediction support model. The proposed method had three parts, namely, introducing fuzzy membership functions, a decision-tree rule induction technique, and fuzzy inference based on Mamdani's method. Outcomes were compared with neural network, logistic regression, decision tree, and Bayes Net. Chaurasia and Pal [31] offered three popular data mining algorithms: CART (classification and regression tree), ID3 (iterative dichotomized 3), and decision table (DT) for diagnosing heart diseases, and the results presented demonstrated that CART obtained higher accuracy within less time.

Olaniyi et al. [29] used neural network and support vector machine for heart diseases. Their proposed method used multilayer perceptron and demonstrated that SVM produced high accuracy. Yan et al. [32] proposed that multilayer perception with hidden layers is found by a cascade process. For the inductive reasoning of the methods, the proposed method used three assessment procedures, namely, cross-validation, hold out, and five bootstrapping samples for five intervals. Yan et al. [33] utilized multilayer perception for the diagnosis of five different cases of heart disease. The method employed a cascade learning process to find hidden layers and used back propagation for training the datasets. Further improvements to the accuracy were achieved by parameter adjustments. Shouman et al. [34] identified gaps in the research work for heart disease diagnosis. The proposed method applied both single and hybrid data mining techniques to establish baseline accuracy and compared. Based on the research, hybrid classifier produced higher accuracy than a single classifier.

Sartakhti et al. [39] presented a method for diagnosis of hepatitis by novel machine learning methods that hybridize support vector machine and simulated annealing process. The proposed method used two hyperparameters for radial basis function (RBF) kernel: *C* and gamma. For all potential combinations of *C* and gamma interval, *k*-fold cross-validation score had been calculated. Results demonstrated that tuning SVM parameters by simulated annealing increased the accuracy. Çalişir et al. [40] developed the principle component analysis and least square support vector machine (PS-LLSVM). The suggested method was carried out in two steps: (1) the feature extraction from hepatitis disease database and feature reduction by PCA and (2) the reduced features are fed to the LSSVM classifier. Li and Wong [41] proposed C4.5 and PCL classifier. The outcomes were compared between C4.5 (bagging, boosting, and single tree) and PCL, and it was concluded that PCL produced higher accuracy than C4.5 based on their observations.

Weng et al. [42] investigated the performance of different classifiers which predicts Parkinson's disease. The proposed method used an ANN classifier based on the evaluation criteria. Jane et al. [43] proposed a Q-back propagated time delay neural network (Q-BTDNN) classifier. It developed temporal classification models that performed the task of classification and prognostication in clinical decision-making system. It used to feed forward time-delay neural network (TDNN) where training was imparted by a Q-learning-induced back propagation (Q-BP) technique. A 10-fold-cross-validation was employed for assessing the classification model. The results obtained were considered for comparative analysis, and it produced high accuracy. Gürüler [44] described a combination of the *k*-means clustering-based feature weighting (KMCFW) method and a complex-valued artificial neural network (CVANN). The suggested method considered five different evaluation methods. The cluster centers were estimated using the KMC. Results obtained showed very high accuracy.

Bashir et al. [45] presented an ensemble framework for predicting people with diabetes with multilayer classification using enhanced bagging and optimized weighting. The proposed HM-BagMOOV method used KNN approach for missing data imputation and had three layers, namely, layer 1 containing naive Bayes (NB), quadratic discriminant analysis (QDA), linear regression (LR), instance-based learning (IBL), and SVM; layer 2 included ANN and RF; and layer 3 used multilayer weighted bagging prediction. The outcome showed that it produced good accuracy for all datasets. Iyer et al. [46] prescribed a method to diagnose the disease using decision tree and naive Bayes. The proposed method used 10-fold cross-validation. The technique had been further enhanced by using other classifiers and neural network techniques. Choubey and Sanchita [47] used genetic algorithm and multilayer perceptron techniques for the diagnosis of diabetics. The suggested methodology was implemented in two levels where genetic algorithm (GA) was used for feature selection and multilayer perceptron neural network (MLP NN) was used for classification of the selected characteristics. The results produced excellent accuracy that was further increased by considering receiver operating characteristic (ROC).

Kharya [48] used various data mining techniques for the diagnosis and prognosis of cancer. The proposed method used neural network, association rule mining, naïve Bayes, C4.5 decision tree algorithm, and Bayesian networks. The results showed that decision tree produced better accuracy than other classifiers. Chaurasia and Pal [49] investigated the performance of different classification techniques on breast cancer data. The proposed method used three classification techniques, namely, SMO, *k*-nearest neighbor algorithm (IBK), and best first (BF) tree. The results demonstrated that SMO produced higher accuracy than the other two techniques. In this article [50], an expert system (ES) is proposed for clinical diagnosis which is helpful for decision making in primary health care. The ES proposed used a rule-based system to identify several diseases based on clinical test reports.

Alzubaidi et al. studied ovarian cancer well [51]. In this work, features are selected using a hybrid global optimization technique. The hybridization process has involved mutual information, linear discriminate analysis, and genetic algorithm. The performance of the proposed hybrid technique is compared with support vector machine. This hybrid technique has shown significant performance improvements than support vector machine.

Gwak et al. [52] have proposed an ensemble framework for combining various crossover strategies using probability. The performance of this context had tested over 27 benchmark functions. It showed outperformance on eight tough benchmark functions. This ensemble framework further can be efficiently used for feature selection of big datasets.

Hsieh et al. [53] have developed and ensemble machine learning model for diagnosing breast cancer. In this model, information-gain has been adopted for feature selection. The list of classifiers used for developing ensemble classifier is neural fuzzy (NF), *k*-nearest neighbor (KNN), and the quadratic classifier (QC). The performance of ensemble framework is compared with individual classifier performance. The results demonstrate that ensemble framework has shown better performance than single classifier.

Review of existing literature for disease diagnosis techniques with machine learning indicates that there exists a plethora of individual classifiers as well as ensemble techniques. However, from such studies, it was also been conclusively evident that no individual classifier gives high prediction accuracy for different disease datasets. This has led to abundant ensemble classifiers for disease diagnosis, compromising the simplicity that an individual classifier offers. To this end, this paper indulges in designing a hybrid system that focuses on providing generalized performance across a broad range of benchmark datasets. The most significant contribution of the proposed hybrid disease classifiers is that unlike most research works mentioned before that targets a specific disease, this paper validates the efficacy of the proposed hybrid classifiers across six different diseases collected over eleven datasets. For instance, among all heart disease, related diagnosis systems only [33] consider five different datasets for the said disease. Also, there are very few attempts in validating diagnosis efficacy over multiple diseases. Shen et al. [54] and Bashir et al. [14] are few exceptions that validate their results for four and five different diseases, respectively. The proposed classifiers employ novel parameter optimization approaches using a few recent evolutionary algorithms, detailed design of which has been presented in subsequent sections.

## 3. Problem Formulation

In this paper, we deal with classifying data from different disease datasets using a hybrid technique that optimizes the parameters of SVM and MLP classifiers for improved disease prediction. The list of objective functions to be targeted while solving the said classification problem include (i) prediction accuracy, (ii) specificity, and (iii) sensitivity, which has been considered very commonly for this problem in existing literature [55–57]. Each of these objective functions captures some aspect of quality of disease classification. In this sense, the problem studied in this paper is a multiobjective optimization problem.

All the aforementioned measures are computed in terms of the following values: true positive (TP), true negative (TN), false positive (FP), and false negative (FN), and their significance is defined as follows: TP: total number of positives that are correctly identified as positive; TN: total number of negatives that are identified as negatives; FP: total number of negatives that are incorrectly identified as positives; and *FN*: total number of positives that are wrongly identified as negatives.

The objective functions considered for optimization in this work are prediction accuracy (PAC), specificity (SPY), and sensitivity (SEY). To model these functions, two random indicator variables are introduced for all the data objects to compute TP, TN, FP, and FN. These are *X*_*i*1_ and *X*_*i*2_, where these are defined as follows:

(1) $$\begin{array}{c} X_{i1}=I\left\{{CL}_{i}={PC}_{i}=C_{+}\right\};\\ X_{i2}=I\left\{{CL}_{i}={PC}_{i}=C_{-}\right\}, \end{array}$$

where *C*_+_ represents the actual class label is positive (+), *C*_−_ represents the actual class label is negative (−), PC_*i*_ represents predicted class label of *i*th data object, and CL*_i_* represents the actual class label of the *i*th data object. At any point of the time, the sum of the entire indicator random variable values is equal to 1; that is, ∑_*j*=1_^2^*X*_*ij*_ = 1, ∀*i*.

Let the classifier being developed for classifying a given dataset be a binary classifier and the dataset has *N* instances with *m*_1_ positive and *m*_2_ negative instances. Therefore,

(2) $$\begin{array}{c} TP=\overset{N}{\underset{i=1}{\sum }}X_{i1},\\ TN=\overset{N}{\underset{i=1}{\sum }}X_{i2},\\ FN=m_{1}-TP=m_{1}-\overset{N}{\underset{i=1}{\sum }}X_{i1},\\ FP=m_{2}{-TN=m}_{2}-\overset{N}{\underset{i=1}{\sum }}X_{i2}. \end{array}$$

The performance parameters for the classifiers can thus be obtained using the following three equations:

(3) $$\begin{array}{c} Prediction\;accuracy\;\left(PAC\right)=\frac{TP+TN}{TP+TN+FP+FN}=\frac{\sum_{i=1}^{N}X_{i1}+\sum_{i=1}^{N}X_{i2}}{m_{1}+m_{2}}, \end{array}$$

(4) $$\begin{array}{c} Specificity\;\left(SPY\right)=\frac{TN}{TN+FP}=\frac{\sum_{i=1}^{N}X_{i2}}{m_{2}}, \end{array}$$

(5) $$\begin{array}{c} Sensitivity\;\left(SEY\right)=\frac{TP}{TP+FN}=\frac{\sum_{i=1}^{N}X_{i1}}{m_{1}}. \end{array}$$

The aim of this research is to arrive at optimal values of classifier parameters through evolution such that some maxima are attained for PAC, SPY, and SEY. It is worthwhile to mention that even different sets of classifier parameter values with same PAC can have different values for SPY and SEY. Thus, there exist tradeoffs among (3), (4), and (5).

Any multiobjective optimization problem can then be solved either by converting the objective functions into a single linear or nonlinear objective function or by computing Pareto fronts using the concept of nondominance [58].

In this paper, a linear combination of objective functions has been taken to form a single linear compound objective function due to the requirement of additional computational effort for finding Pareto fronts in every iteration.

(6) $$\begin{array}{c} Maximize\;Z=W_{1}*PAC+W_{2}*SPY+W_{3}*SEY,\\ Maximize\;Z=W_{1}*\frac{\sum_{i=1}^{N}X_{i1}+\sum_{i=1}^{N}X_{i2}}{m_{1}+m_{2}}+W_{2}*\frac{\sum_{i=1}^{N}X_{i2}}{m_{2}}+W_{3}*\frac{\sum_{i=1}^{N}X_{i1}}{m_{1}} \end{array}$$

subject to the constraints

(7) $$\begin{array}{c} W_{1}+W_{2}+W_{3}=1, \end{array}$$

(8) $$\begin{array}{c} 1\geq W_{i}\geq 0\;\forall i, \end{array}$$

(9) $$\begin{array}{c} U_{i}\geq {CLASSIFIER\_PAR}_{i}\geq L_{i}\;\forall i, \end{array}$$

where CLASSIFIER_PAR_*i*_ is the *i*th sensitive parameter of the considered classifier, (7) represents the totality condition of the weights, (8) guarantees the nonnegativity condition, and (9) checks that the *i*th classifier parameter values is within the specified bounds.

## 4. Evolutionary Algorithms

In this section, we present a summary of the three evolutionary algorithms employed to optimize the parameters of SVM and MLP for classifying medical datasets for disease diagnosis. The discussions are restricted only to provide a brief overview. Detailed information and possible variations of these algorithms are beyond the scope of this paper.

### 4.1. Gravitational Search Algorithm (GSA)

Gravitational search algorithm (GSA) is one of the population-based stochastic search methods initially developed by Rashedi et al. in the year 2009 [59]. GSA is inspired by Newton's gravitational law in physics, where every particle in the universe attracts every other particle with a force that is directly proportional to the product of their masses and inversely proportional to the square of the distance between them. GSA has been successfully applied to solve several engineering optimization problems [60, 61].

In GSA, several masses are considered on a *d*-dimensional space. The position of each mass resembles a point in the solution space of the problem to be solved. The fitness values of the agent, worst (*t*), and best (*t*) are used to compute the force (*F*) of mass. Equations corresponding to these parameters are provided in

(10) $$\begin{array}{c} q_{i}\left(t\right)=\frac{{fit}_{i}\left(t\right)-worst\left(t\right)}{best\left(t\right)-worst\left(t\right)}, \end{array}$$

(11) $$\begin{array}{c} M_{i}\left(t\right)=\frac{q_{i}\left(t\right)}{\sum_{j=1}^{s}q_{j}\left(t\right)}, \end{array}$$

(12) $$\begin{array}{c} best\left(t\right)=\min\left\{{fit}_{k}\left(t\right):\forall k\right\}, \end{array}$$

(13) $$\begin{array}{c} worst\left(t\right)=\max\left\{{fit}_{k}\left(t\right):\forall k\right\}. \end{array}$$

To update the position of mass (*x*_*i*_^*d*^(*t* + 1)), velocity (*v*_*i*_^*d*^(*t*)) needs to be updated first. The velocity of the mass at the time (*t* + 1) majorly depends on the values of velocity and acceleration at that time instant *t*. Acceleration of the *i*th mass at instant *t* is (*a*_*i*_^*d*^(*t*)) depending on forces of all other heavy masses based on (14). The equation corresponding to the acceleration is given in (15). Equations corresponding to updating process of mass position and mass velocity are provided in (16) and (17).

(14) $$\begin{array}{c} F_{i}^{d}\left(t\right)=\sum_{j\in kbest,j\neq i}{\mathrm{rand}}_{j}\left(G\left(t\right)\frac{M_{j}\left(t\right)M_{i}\left(t\right)}{R_{ij}\left(t\right)+\in }\left(x_{j}^{d}\left(t\right)-x_{i}^{d}\left(t\right)\right)\right), \end{array}$$

(15) $$\begin{array}{c} a_{i}^{d}\left(t\right)=\frac{F_{i}^{d}\left(t\right)}{M_{i}\left(t\right)}, \end{array}$$

(16) $$\begin{array}{c} V_{i}^{d}\left(t+1\right)={\mathrm{rand}}_{i}\times V_{i}^{d}\left(t\right)+a_{i}^{d}\left(t\right), \end{array}$$

(17) $$\begin{array}{c} X_{i}^{d}\left(t+1\right)=X_{i}^{d}\left(t\right)+V_{i}^{d}\left(t+1\right), \end{array}$$

where rand_*i*_ and rand_*j*_ lie between 0 and 1. “∈” is a small value. The distance between agents *i* and *j* is denoted by *R*_*ij*_(*t*). The best *k* agents are denoted with *k*best. *G* is a gravitational constant which is initialized with *G*_0_ at the beginning, and with the progress in time, the value of *G* decreases.

### 4.2. Particle Swarm Optimization (PSO)

In 1995, Dr. Kennedy and Dr. Eberhart developed a population-based speculative computational optimization procedure called particle swarm optimization based on the social behavior of living organisms like fish schools and bird flocks [62]. In PSO, the particles are randomly initialized. Position and velocity of the particles are represented as *X*_*i*_ and *V*_*i*_, respectively. The fitness function is computed for each particle. Personal best (pBest) and global best (gBest) are the two important factors in PSO. Each particle has its own personal best, which is the particles' individual best so far achieved until a time instant *t*. Global best is the overall best of all particles upto the time instant *t*. The algorithm is executed for a certain number of iterations. At each iteration, velocity is updated for all particles using a velocity updating scheme [63] as depicted in

(18) $$\begin{array}{c} V_{id}\left(t\right)=w*V_{id}\left(t-1\right)+c_{1}*\mathrm{rand}\left(\right)*\left({pBest}_{id}-X_{id}\left(t-1\right)\right)+c_{2}*\mathrm{rand}\left(\right)*\left({gBest}_{id}-X_{id}\left(t-1\right)\right), \end{array}$$

where *w* represents the inertia weight, *c*_1_ and *c*_2_ are the personal and global learning factors, and rand() is a random number between [0,1].

The following equation updates the new position of the particle:

(19) $$\begin{array}{c} X_{i}^{d}\left(t\right)=X_{i}^{d}\left(t-1\right)+V_{i}^{d}\left(t\right). \end{array}$$

The basic steps of PSO are given in Algorithm 1.

### 4.3. Firefly Algorithm (FA)

The firefly algorithm is a recently proposed bioinspired, evolutionary metaheuristic that mimics the social behavior of firefly species. Fireflies produce short and rhythmic flashes, the pattern of which characterizes particular species. The artificial, firefly-inspired algorithm makes certain assumptions regarding its functioning, such as unisexual fireflies for ensuring that all artificial fireflies attract each other and that the attractiveness is proportional to their brightness to define the potential of relative firefly movements. The brightness of a firefly is defined based on the problem at hand that it needs to optimize. For the minimization problem, the brightness may be the reciprocal of the objective function value. The pseudocode of the basic firefly algorithm as given by Yang in [64] has been depicted in Algorithm 2. The list of equations used in firefly algorithm is given as follows:

(20) $$\begin{array}{c} X_{i}^{t+1}=X_{i}^{t}+V_{i}^{t}, \end{array}$$

(21) $$\begin{array}{c} V_{i}^{t}=\beta_{0}e^{-ϒr^{2}}d\left(X_{i}-X_{j}\right)+\alpha \left(\mathrm{rand}-0.5\right), \end{array}$$

(22) $$\begin{array}{c} \begin{array}{c} r_{ij}=d\left(X_{i}-X_{j}\right)=\left\|X_{i}-X_{j}\right\|=Euclidean\;distance\;between\;X_{i}\;and\;X_{j},\\ \beta \left(r\right)=\beta_{0}e^{-ϒr^{m}}\;where\;m\geq 1, \end{array} \end{array}$$

where *β*_0_, *ϒ*, *α* ∈ [0, 1].

Each firefly position is updated based on (20). The velocity of *i*th firefly is based on a fraction of attractiveness in the distance between fireflies *X*_*i*_ and *X*_*j*_ in an *m*-dimensional space and also on *α*, a small random value in the range 0 to 0.2; the equations related to velocity are given in (21) and (22).

## 5. Classification Techniques

Two classification techniques are used, and the basic details of these techniques are discussed in the subsequent sections.

### 5.1. Multilayer Perceptron

Multilayer perceptron (MLP) is the most commonly used supervised feed forward artificial neural network. It is a modification of the linear perceptron algorithm. It consists of many nodes that are arranged in several layers. In general, MLP contains three or more processing layers: one input layer for receiving input features, one or more hidden layers, and an output layer for producing classification results based on the classes [65].

Each node is represented as an artificial neuron which converts the input features into output using a weighted sum of inputs and activation function.

The weighted input is given by

(23) $$\begin{array}{c} V_{i}=\sum W_{ij}X_{i}+\Theta_{i},\\ Y_{i}=f_{i}\left(V_{i}\right), \end{array}$$

where *V* is the weighted sum of input features, *W* represents weights, *X* represents the input features, and Θ is the bias based on the classes.

The activation function is denoted by *f*(*x*). The most frequently used activation functions are sigmoids. They are as follows:

(24) $$\begin{array}{c} \begin{array}{c} f\left(v_{i}\right)=\tanh\left(v_{i}\right),\\ f\left(v_{i}\right)={\left(1+e^{-vi}\right)}^{-1}. \end{array} \end{array}$$

The multilayer perceptron is trained using back propagation (BP). The weight update equation used in BP is given in

(25) $$\begin{array}{c} \begin{array}{cc} w_{ji}\leftarrow w_{ji}+{\eta \delta }_{j}\;x_{ji}+\alpha ∆w_{ij}\left(n-1\right) & where\;1\geq \eta ,\alpha \geq 0. \end{array} \end{array}$$

The parameter's learning rate (*η*) and momentum (*α*) are evolved using evolutionary algorithms presented in Section 4. A very basic MLP algorithm is provided in Algorithm 3 [66, 67].

There is a chance of being caught at a local minimum during the process of back propagation learning, and hence, to overcome it in this research article, learning rate and momentum values are evolved using 3 evolutionary search algorithms (CSO, IWO, and FF). A 3-layer neural network has been executed with an input layer, one hidden layer, and an output layer. The size of the input layer is equal to the number features of the data, and also, the size of the output layer is nothing but the number classes. The size of the hidden layer is the average of input and output layer sizes. Moreover, the performance of these three algorithms is compared in the simulation and is discussed in the Results and Discussion of this paper.

### 5.2. Support Vector Machine

Support vector machine (SVM) is one of the supervised machine learning algorithms, which are often used for binary classifications. It was originally developed by Vapnik in 1979 [68]. The training data is in the form of instance-value pairs (*x*_*i*_, *y_i_*). The SVM classifier finds an optimal hyperplane to separate negative and positive classes, and it is represented by *F*(*x*) = *w*^*t*^ · *x* + *b* = 0.

Based on the class labels, two hyperplanes are formed, which are as follows:

*F*(*x*) = *w*^*t*^ · *x* + *b* ≥ 0 for positive instances (*y*_*j*_ = +1) and *F*(*x*) = *w*^*t*^ · *x* + *b* ≤ 0 for negative instances (*y*_*j*_ = −1), where *w* is the weight vector, *x* is input vector, and *b* is bias. Classifications are made on the hyperplanes thus formed.

The optimization problem formed during the development of soft margin classifier is as follows:

(26) $$\begin{array}{c} Minimize⌻Z=\frac{1}{2}<w,w>+\;C\sum_{i}\xi_{i},\\ subject\;to⌻Y_{i}\;\left(<w_{i},x_{i}>+b\right)\geq \left(1-\xi_{i}\right). \end{array}$$

The parameter cost (*C*) mentioned in (26) will be evolved using the evolutionary algorithms mentioned in Section 4.

## 6. Hybrid Intelligent System for Diagnosing Diseases

Diagnosing diseases from data collected from many patients with a varied degree of a specific disease is a classification problem. In medical information systems, single classifiers as well as ensemble classifiers have been studied for the disease diagnosis problem. In this section, we present the design of hybrid systems that employ evolutionary algorithms as well as classification techniques to classify diseases based on data. A few hybrid systems have been developed to optimize the parameters of the classifiers [69, 70]; however, the premises of such classifiers are different application domains.

**Figure 1:**
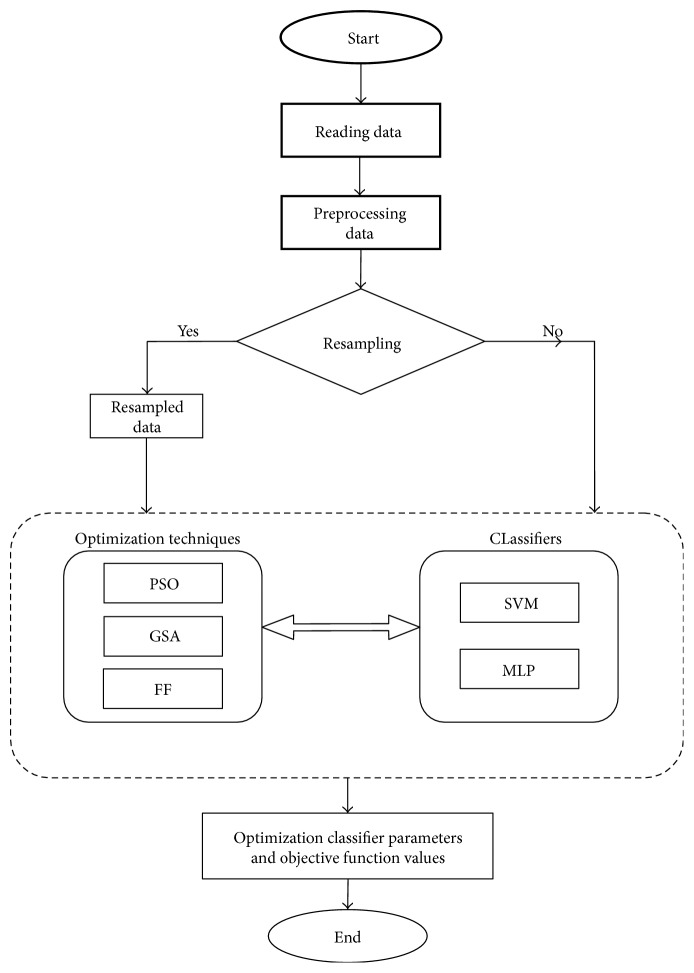
Schematic representing the flow of steps in the proposed hybrid disease diagnosis system.

The performance of any classifier broadly depends on three factors, namely, the technique used for the classification; data statistics (regression, entropy, kurtosis, standard deviation, number of features considered for training, size of the training data, etc.); and parameters of the classifier (learning rate, depth of the tree, maximum number of child nodes allowed for a parent node in the decision tree, pruning, fuzzy membership functions, activation functions, etc.). In this paper, we focus on optimizing the parameter classifiers using evolutionary algorithms, and thus, our designed system qualifies as a hybrid system.  Figure 1 illustrates a schematic block diagram of the proposed hybrid system depicting the major steps to be carried out to arrive at disease diagnosis. The rectangle with dotted border illustrates the main emphasis of this paper. It represents that in this paper, we have studied how two classifiers, namely, SVM and MLP, perform as far as disease diagnosis is concerned. The parameters of these two classifiers have been optimized using three evolutionary algorithms, namely, PSO, GSA, and FA, with the goal of maximizing quality of diagnosis in terms of PAC, SPY, and SEY; or simply said, the goal is to optimize the three objectives as has been explained in Section 3. This has been depicted in the left half of the dotted rectangle in Figure 1. The basic steps involved in the hybrid system are summarized in Algorithm 4.

Preprocessing stage handles missing data of a feature by inserting most popular data or interval estimated data for that feature. As a part of preprocessing, features have also been normalized using min–max norm with the goal of reducing training phase time of classifiers, which takes quite some time due to the varied range of the feature values. In step 3, we have employed two classifiers (SVM and MLP). In step 4, the parameters selected for evolving in SVM are COST whereas for MLP, two parameters, namely, learning rate and momentum, have been selected for evolution. The range of these three parameters (cost, learning rate, and momentum) has been set as [0, 1]. In step 5, the objective function selected is either a single objective or multiobjective. If the method of optimization is multiobjective optimization, then for the sake of simplicity or uniformity, convert all the objective functions into either maximization or minimization. The multiple objectives considered for multiobjective optimization are given in (3)–(9). In step 6, three evolutionary algorithms (cat swarm optimization, gravitational search algorithm, and firefly algorithm) are selected as optimization techniques to find the optimum parameter values for the considered classifiers with respective to the multiple objectives: prediction accuracy, sensitivity, and specificity. Equations corresponding to multiobjective optimization are given in (3)–(9). In step 8, postprocessing of the results found in step 7 has to be done based on the optimization model selected in step 5. If the optimization model is single objective optimization, then to check the performance of the evolutionary algorithm, several statistical values like max, minimum, mean, median, and so forth have to be computed. If the selected optimization model is multiobjective (or weighted multiobjective) optimization model, the quality of nondominated solutions must found using the metrics like spacing, generational distance, and so forth.

The hybridization process ensures that the population of the evolutionary algorithms is constructed based on the classifier parameters by satisfying parameter bounds. During the execution of evolutionary algorithms, population fitness is computed by substituting the performance parameter values of the classifier executed on the dataset in step 4.

Once all the three EAs are executed individually, optimal parameter values for each of the two classifiers (SVM and MLP) are found, and subsequently, these six HISs are compared based on their fitness values. That HIS having the best fitness value for a particular dataset is considered as the proposed HIS for that particular dataset. The objective function and parameter values of the best hybrid intelligent system are treated as final optimal values.

By combining the two classifiers and the three evolutionary optimization techniques for optimizing chosen classifier parameters, a number of hybrid intelligent systems have been obtained as possible alternatives. These alternative hybrid intelligent systems (HISs) have been termed as GSA-based SVM (GSVM), FA-based SVM (FSVM), PSO-based SVM (PSVM), GSA-based MLP (GMLP), FA-based MLP (FMLP), and PSO-based MLP (PMLP). These six HISs are tested on all the eleven benchmark datasets considered in this work, once without employing resampling and then using resampling technique. Hence, these HISs produce a set of sixteen results for each of the disease datasets, eight for SVM and eight for MLP. Out of these eight results, one is for the basic classifier (only SVM and only MLP) without data resampling, another for the same with resampling data, and the remaining six are for the three evolutionary algorithms each, once with original data and again with resampling data. The benchmark datasets are tested with ADABOOST version of SVM and MLP. However, on average, the ADABOOST results are not competitive with the instance-based supervised resampling technique in Weka, and the corresponding performances are given in Table 1. Moreover, we continued our experiments using instance-based supervised resampling technique.

## 7. Simulations and Results

To check the performance of the proposed hybrid system, 11 medical datasets of various diseases are considered. These data have been collected from the UCI repository [71], and the same form the basis of almost all performance evaluations in disease diagnosis. A detailed account of the datasets employed in this paper has been summarized in Table 2. All the six hybrid system alternatives and basic classifier technique have been executed on each of the 11 datasets, once without resampling of the dataset and then repeated with resampled dataset.

**Figure 2:**
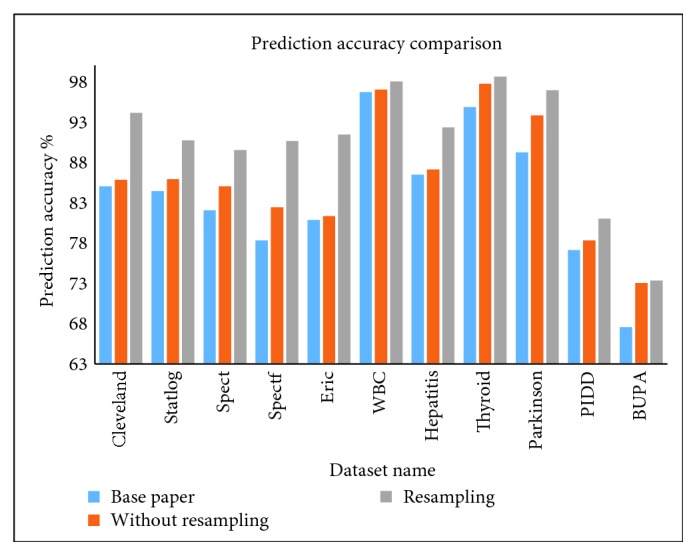
Comparison of all techniques based on prediction accuracy.

All the three evolutionary algorithms are executed for 50 iterations by considering 20 agents per iteration. These algorithms have been implemented in Java. Weka 3.7.4 tool class libraries have been used for the implementation of SVM and MLP. Instance-based resampling, which is available in Weka, has been used for resampling purposes. For experimentation purposes, the datasets considered are divided into testing and training sets, and a 10-fold cross-validation is used to that effect. To compare the performance of our proposed hybrid system for the datasets employed, we have compared results we have obtained with the results presented in three very recent papers that use the same datasets (not all 11 datasets, only a subset is utilized by these papers). References [14] through [16] are recent literature, and they have been referred to in our work as the *base papers* for every dataset (as has been earmarked in legends in Figure 2). The results of datasets corresponding to diseases like breast cancer, hepatitis, BUPA liver, Pima, Cleveland, and Parkinson have been compared with those of [14], whereas the results of Statlog, Spect, Spectf, and Eric have been compared with those of BagMOOV [15]. Thyroid disease results alone are compared with those of [16]. In this work, the highest priority is given in favor of prediction accuracy. Hence, *w*_1_, *w*_2_, and *w*_3_ in (6) correspond to 0.95, 0.05/2, and 0.05/2, respectively.

### 7.1. Statistical Analysis

In this section, we present a number of statistical analyses for the results obtained from our proposed hybrid system. The following subsections provide details about how these analyses are done.

#### 7.1.1. Signed-Rank Test

The statistical analysis was done using Wilcoxon signed-rank test [72]. It tests the performance of all the techniques. The null hypothesis and alternative null hypothesis are set as follows:

 
*H*_0_: median(X) is equal to median(Y).
 
*H*_1_: median(X) is not equal to median(Y).

The objective values corresponding to FMLP overall disease datasets are tested over rest of the five techniques on all disease datasets, once with and next without resampling for each of the hybrid system alternatives, namely, FSVM, GSVM, PSVM, GMLP, and PMLP.

The Wilcoxon signed-rank test was executed with the level of significances 0.01 and 0.05. The Matlab function “signrank()” was used to perform the statistical analysis and the conclusions arrived upon has been presented in Tables 3, 4, 5, and 6.

#### 7.1.2. Student's *t*-Test

Student's *t*-test is used to test whether the sample *X* derived from a normal distribution can have the mean *m* without knowing standard deviation [73]. We execute FMLP for 20 times, and we also noted the best performance in each iteration. Student's *t*-test is executed on the three objectives: prediction accuracy (PAC), sensitivity (SEN), and specificity (SPE). Null hypothesis and alternative hypothesis are set as follows:

 
*H*_0_: *μ*_*X*_ = *m*, *H*_1_: *μ*_*X*_ ≠ *m*.

Student's *t*-test is performed by using ttest() function available in MATLAB. The performance of this test for various parameter values has been summarized in Tables 7 and 8.

### 7.2. Performance Metrics

The important distinct goals of multiobjective optimization are (1) finding solutions as close to the Pareto-optimal solutions as possible and (2) finding solutions as diverse as possible in the obtained nondominated front. In this work, to test the first goal is tested using generational distance (GD) and the second target is tested by computing spacing [58]. In the metric computation, two sets are used, namely, *Q* and *P*^∗^, where *Q* is the Pareto front found by test algorithm and *P*^∗^ is the subset of true Pareto-optimal members. Before computing these metrics, the data in *Q* is to be normalized since various objective functions will have different ranges.

Generational distance (GD): Veldhuizen introduces this metric in the year 1990 [74]. This metric finds an average distance between the members of *Q* and *P*^∗^ as follows: GD = (∑_*i*=1_^|*Q*|^*d*_*i*_^*p*^)^1/*p*^/|*Q*|. For *p* = 2, the parameter *d_i_* is the Euclidean distance between the members of *Q* and the nearest member of *P*^∗^: $d_{i}=\min_{k=1}^{\left|P^{*}\right|}\sqrt{\sum_{m=1}^{M}{\left(f_{m}^{i}-f_{m}^{*\left(k\right)}\right)}^{2}},$ where *f*_*m*_^∗(*k*)^ is the *m*th objective function value of the *k*th member of *P*^∗^. An algorithm having a small value of GD is better. The members in *P*^∗^ are having a maximum value for at least one objective function.

Spacing (SP): Schott introduces this metric in the year 1995 [75]. This metric finds the standard deviation of different *d*_*i*_ values. It can be calculated as follows: $S=\sqrt{\left(1/\left|Q\right|\right)\sum_{i=1}^{\left|Q\right|}{\left(d_{i}-\bar{d}\right)}^{2}},$ where *d*_*i*_ = min_*k*∈*Q*∩*k*≠i_∑_*m*=1_^*M*^|*f*_*m*_^*i*^ − *f*_*m*_^*k*^| and $\bar{d}$ is the mean value of *d*_*i*_'s. A good algorithm will be having a minimal SP value. The set *Q* is caught by executing FMLP for 50 iterations with each iteration having 20 agents. In every iteration, the Pareto fronts are stored in external memory. The metrics for GD and SP for all the three objectives with and without resampling are given in Table 9.

### 7.3. Results and Discussion

The best values found in all the hybrid systems are discussed as follows.

#### 7.3.1. Cleveland Dataset

The performance of all the 8 techniques (2 basic machine learning and six hybrid systems) over Cleveland dataset is depicted in Table 10. PMLP shows best sensitivity (84.79%), whereas FMLP shows better results for all the other performance parameters, like accuracy (85.8%), specificity (87.5%), *F*-measure (85.74%), recall (85.8%), and precision (85.91%) without resampling. On the contrary, with resampling, PMLP shows the best accuracy (94.1%), but for all the other parameters, like sensitivity (93.49%), specificity (94.77%), *F*-measure (94.05%), recall (94.05%), and precision (94.07%), PMLP (GMLP, FMLP) shows best results. A comparison of Cleveland result with the state-of-the-art result is given in Table 11. Table 10 summarizes the performance of the proposed hybrid alternatives for the Cleveland dataset, and Table 11 compares this performance with best results obtained in recent literature.

#### 7.3.2. Statlog Dataset

The performance of all the 8 techniques (2 machine learning and six hybrid systems) over Statlog dataset with and without resampling is given in Table 12. The highest accuracy, sensitivity, specificity, *F*-measure, recall, and precision without resampling are achieved by FMLP, PMLP, FMLP, FMLP, FMLP, and FMLP, respectively; best values found have been bolded for easy identification in Table 12. The highest accuracy, sensitivity, specificity, *F*-measure, recall, and precision with resampling are achieved by GMLP, GMLP, FMLP, GMLP, GMLP, and GMLP, respectively; best values found have been bolded for easy identification in Table 12. A comparison of Statlog result with the state-of-the-art result is given in Table 13. The highest prediction accuracy for Statlog is 85.9% (without resampling) and 90.7 (with resampling). The performance of all the considered techniques over Statlog dataset with resampling is better than without resampling. Table 12 summarizes the performance of proposed hybrid alternatives for this dataset, and Table 13 compares this performance with best results obtained in recent literature.

#### 7.3.3. Spect Dataset

The performance of all the 8 techniques (2 machine learning and six hybrid systems) over Spect dataset with and without resampling is given in Table 14. The highest accuracy, sensitivity, specificity, *F*-measure, recall, and precision without resampling are achieved by FMLP, FSVM, FMLP, FMLP, FMLP, and FMLP, respectively, with the values 85%, 88.4%, 74.2%, 83.3%, 85%, and 83.9%. The highest accuracy, sensitivity, specificity, *F*-measure, recall, and precision with resampling are achieved by GMLP (PMLP), GMLP (FMLP, PMLP), GMLP (PMLP), PMLP (GMLP), GMLP (PMLP), and GMLP (PMLP), respectively, with the values 89.5%, 91.9%, 77.3%, 89.2%, 89.5%, and 89.1%. A comparison of Spect result with the state-of-the-art result is given in Table 15. The highest prediction accuracy for Spect is 85% (without resampling) and 89.5 (with resampling). The performance of all the considered techniques over Spect dataset with resampling is better than without resampling. Table 14 summarizes the performance of proposed hybrid alternatives for this dataset, and Table 15 compares this performance with best results obtained in recent literature.

#### 7.3.4. Spectf Dataset

The performance of all the 8 techniques (2 machine learning and six hybrid systems) over Spectf dataset with and without resampling is given in Table 16. The highest accuracy, sensitivity, specificity, *F*-measure, recall, and precision without resampling are achieved by FMLP, FSVM, FMLP, PSVM, FMLP, and FMLP, respectively, with the values 82.4%, 88%, 83.3%, 80.6%, 82.4%, and 82.6%. The highest accuracy, sensitivity, specificity, *F*-measure, recall, and precision with resampling are achieved by PMLP, GSVM, PMLP, PMLP, PMLP, and PMLP, respectively; best values found are bolded for easy identification in Table 16. A comparison of Spectf result with the state-of-the-art result is given in Table 17. The highest prediction accuracy for Spectf is 82.4% (without resampling) and 90.6% (with resampling). The performance of all the considered techniques over Spectf dataset with resampling is better than without resampling except in specificity. Table 16 summarizes the performance of proposed hybrid alternatives for this dataset, and Table 17 compares this performance with best results obtained in recent literature.

#### 7.3.5. Eric Dataset

The performance of all the 8 techniques (2 basic machine learning and six hybrid systems) over ERIC dataset is depicted in Table 18. FMLP shows best results for parameters like accuracy (81.34%), specificity (79.1%), *F*-measure (81.02%), and recall (81.34%), whereas GMLP shows better results for sensitivity (88.41%) and precision (82.5%) without resampling. On the contrary, with resampling, GMLP shows best results for parameters like accuracy (91.39%), sensitivity (88.78%), specificity (93.69%), *F*-measure (91.40%), recall (91.39%), and precision (91.48%). A comparison of ERIC result with the state-of-the-art result is given in Table 19. Table 18 summarizes the performance of the proposed hybrid alternatives for the ERIC dataset, and Table 19 compares this performance with best performance in recent literature.

#### 7.3.6. Wisconsin Breast Cancer (WBC) Dataset

The performance of all the 8 techniques (2 basic machine learning and six hybrid systems) over breast cancer dataset is depicted in Table 20. GMLP shows best accuracy (97%) and precision (97.04%), whereas PSVM shows better results for the parameters like sensitivity (95.08%), specificity (98.02%), and *F*-measure (97%), and GMLP and PSVM together show the best result for recall (97%) without resampling. On the contrary, with resampling, FMLP shows the best accuracy (98%), but for all the other parameters, like sensitivity (96.61%), *F*-measure (98%), recall (98%), and precision (98%), PMLP (FMLP) shows best results and PSVM (GSVM and FSVM) shows best results for specificity (99.55%). A comparison of breast cancer result with the state-of-the-art result is given in Table 21. Table 20 summarizes the performance of the proposed hybrid alternatives for the breast cancer dataset, and Table 21 compares this performance with best results obtained in recent literature.

#### 7.3.7. Hepatitis Dataset

The performance of all the 8 techniques (2 basic machine learning and six hybrid systems) over Hepatitis dataset is depicted in Table 22. PMLP shows best results for specificity (90.55%), *F*-measure (86.77%), recall (87.1%), and precision (86.6%), whereas GSVM (PSVM and FSVM) shows better results for the parameters like accuracy (87.1%) and sensitivity (73.08%) without resampling. On the contrary, with resampling, FMLP (PMLP and GMLP) shows best results for parameters like accuracy (92.26%), sensitivity (80.77%), specificity (94.57%), *F*-measure (92.14%), recall (92.26%), and precision (92.08%). A comparison of Hepatitis result with the state-of-the-art result is given in Table 23. Table 22 summarizes the performance of the proposed hybrid alternatives for the Hepatitis dataset, and Table 23 compares this performance with best results obtained in recent literature.

#### 7.3.8. Thyroid Dataset

The performance of all the 8 techniques (2 machine learning and 6 hybrid systems) over thyroid dataset with and without resampling is given in Table 24. The highest accuracy, sensitivity, specificity, *F*-measure, recall, and precision without resampling is achieved by FMLP, FMLP (PMLP), FMLP, FMLP, FMLP (PMLP), and FMLP (PMLP), respectively, best values found have been bolded for easy identification in Table 24. The highest accuracy, sensitivity, specificity, *F*-measure, recall, and precision with resampling are achieved by PMLP (fMLP), FMLP (PMLP), PMLP (FMLP), PMLP (FMLP), PMLP (FMLP), and PMLP (FMLP), respectively, with the values 98.6%, 98.2%, 98.74%, 98.6%, 98.6%, and 98.6%. A comparison of thyroid result with the state-of-the-art result is given in Table 25. The highest prediction accuracy for thyroid is 97.7% (without resampling) and 98.6% (with resampling). The performance of all the considered techniques over thyroid dataset with resampling is better than without resampling. Table 24 summarizes the performance of proposed hybrid alternatives for this dataset, and Table 25 compares this performance with best results obtained in recent literature.

#### 7.3.9. Parkinson Dataset

The performance of all the 8 techniques (2 machine learning and six hybrid systems) over Parkinson dataset with and without resampling is given in Table 26. The highest accuracy, sensitivity, specificity, *F*-measure, recall, and precision without resampling are achieved by FMLP, FMLP, PSVM (FSVM, GSVM), FMLP, FMLP, and FMLP, respectively, with the values 93.8%, 96.6%, 96.2%, 93.9%, 93.8%, and 94%. The highest accuracy, sensitivity, specificity, *F*-measure, recall, and precision with resampling are achieved by GMLP, GMLP (FMLP, PMLP), PSVM (GSVM, FSVM), PMLP (GMLP), PMLP (GMLP), and PMLP (GMLP), respectively, with the values 96.9%, 97.4%, 100%, 96.9%, 96.9%, and 96.9%. A comparison of Parkinson result with the state-of-the-art result is given in Table 27. The highest prediction accuracy for Parkinson is 93.8% (without resampling) and 96.9% (with resampling). The performance of all the considered techniques over Pakinson dataset with resampling is better than without resampling. Table 26 summarizes the performance of proposed hybrid alternatives for this dataset, and Table 27 compares this performance with best results obtained in recent literature.

#### 7.3.10. Pima Indian Diabetic Dataset

The performance of all the 8 techniques (2 machine learning and six hybrid systems) over Pima dataset with and without resampling is given in Table 28. The highest accuracy, sensitivity, specificity, *F*-measure, recall, and precision without resampling are achieved by FMLP, FMLP, PSVM (FSVM, GSVM), FMLP, FMLP, and FMLP, respectively; best values found have been bolded for easy identification in Table 28. The highest accuracy, sensitivity, specificity, *F*-measure, recall and precision with resampling are achieved by FMLP, GMLP, FMLP, FMLP, FMLP, and FMLP, respectively; best values found have been bolded for easy identification in Table 28. A comparison of Pima result with the state-of-the-art result is given in Table 29. The highest prediction accuracy for Pima is 78.3% (without resampling) and 81% (with resampling). The performance of all the considered techniques over Pima dataset with resampling is better than without resampling. Table 28 summarizes the performance of proposed hybrid alternatives for this dataset, and Table 29 compares this performance with best results obtained in recent literature.

#### 7.3.11. BUPA Liver Disease Dataset

The performance of all the 8 techniques (2 machine learning and six hybrid systems) over BUPA dataset with and without resampling is given in Table 30. The highest accuracy, sensitivity, specificity, *F*-measure, recall, and precision without resampling are achieved by GMLP, PSVM, FMLP, FMLP, FMLP, and FMLP, respectively, with the values 73%, 72%, 74.9%, 72.8%, 73%, and 72.8%. The highest accuracy, sensitivity, specificity, *F*-measure, recall, and precision with resampling are achieved by GMLP, FMLP, GMLP, GMLP, GMLP, and GMLP, respectively, with the values 73.3%, 67.5%, 78.4%, 73.3%, 73.3%, and 73.9%. A comparison of BUPA result with the state-of-the-art result is given in Table 31. The highest prediction accuracy for BUPA is 73% (without resampling) and 73.3% (with resampling). The performance of all the considered techniques over BUPA dataset with resampling is better than without resampling except in sensitivity. Table 30 summarizes the performance of proposed hybrid alternatives for this dataset, and Table 31 compares this performance with best results obtained in recent literature.

In GSA updating of an agent, the position is learned from all other agents, whereas in PSO updating of an agent position is based on two parameters called gBEST and pBEST. In each iteration of these two algorithms at most *n*, new solutions are brought forth. However, in FA in the worst case, each agent develops *O*(*n*) new solution by moving towards all other best solutions. Therefore, in the worst case of FA, space is managed more efficiently than the other two algorithms (GSA and PSO). The same is demonstrated over the 11 medical datasets.

From the previous observations, it is concluded that MLP without resampling shows improvement in all datasets when compared with latest literature results and the same is depicted in Figure 2. As mentioned earlier, in Figure 2, the blue bar represents best performance in literature. Best results obtained by any of the six HISs proposed in this paper has been depicted in Figure 2 alongside, once without resampling (orange bar) and then with resampled data (gray bar). Sensitivity and specificity values for all systems have been presented in Tables 32 and 33, and it can be observed that our proposed hybrid system performs very well across all the datasets, in particular, the parameter optimized MLP.  Table 34 summarizes the optimal parameter values of MLP. Hence, in comparison with the ensemble techniques, parameter optimized MLP gives a better result.

Table 3 gives the outcomes of the rank test for the results of with resampling at the level of significance (LOS) 0.01. If *h* value is zero, then *H*_0_ is true otherwise *H*_1_ is true. In Table 3, 161 ones are there out of a total of 165. It means that 161 times null hypothesis is false and four times null hypothesis is true.

Table 4 gives the outcomes of the rank test for the results of with resampling at the level of significance (LOS) 0.05. In Table 4, 163 ones are there out of 165. It means that 163 times null-hypothesis is false and four times null hypothesis is true.

Table 5 gives the outcomes of the rank test for the results of without resampling at the level of significance (LOS) 0.01. In Table 5, 164 ones are there out of 165. It means that 164 times null hypothesis is false and one time null hypothesis is true.

Table 6 gives the outcomes of the rank test for the results of without resampling at the level of significance (LOS) 0.05. In Table 6, 164 ones are there out of 165. It means that 164 times null hypothesis is false and one-time null hypothesis is true.

The outcomes of FMLP are taken as “*m*” value. Tables 7 and 8 give the results of *t*-test for both resampling and without resampling techniques with LOS 0.01 and 0.05. In these tables, *h* value is zero for all datasets at 0.01 and 0.05 LOS. Hence, null hypothesis is accepted for all datasets.

## 8. Conclusion

Due to the complex framework of ensemble approach and the moderate performance of the individual classifier, hybrid systems have a lot of promise in the diagnosis and prognosis of diseases. To overcome these, we proposed a disease diagnosis system by juxtaposing three evolutionary algorithms and SVM and MLP classifiers. Three evolutionary algorithms optimize the parameters of the two classifiers and such enhanced classifiers have been used to train and diagnose diseases. Accordingly, six hybrid diagnosis alternatives have been obtained by working out the combinations of classifiers and evolutionary algorithms. Based on results presented in this paper, it can be concluded that our hybridization approach provides high prediction accuracy than other methods in literature across a wide variety of disease datasets. Even among the six alternative parameter optimized classifier systems proposed, FMLP was found to be the relatively best across the majority of the 11 datasets considered. On average, MLP shows 2.2% and 6.814% improvement in prediction accuracy on the 11 datasets with and without resampling. The ranges of improvements shown by MLP in the objective sensitivity are −2.9 to 75.13 and −9.68 to 86.33 without and with resampling, respectively. The ranges of improvement shown by MLP in the objective specificity are −9.68 to 86.33 and −18.93 to 36.33 without and with resampling, respectively. From the experimental results, it is concluded that FMLP shows outperformance than recently developed ensemble classifiers ([14, 15]). As a part of the continuation of this research, we intend to process a very higher dimensional dataset with the major phases of feature selection and parameter evolution of the classifier. For feature selection, similarity metric-based hypergraph will be constructed and then by using hypergraph special properties, important topological and geometrical features will be identified. In phase 2, competitive and co-operative parallel hybrid intelligent systems will be employed for incorporating direct and indirect communication among the different systems at guaranteed run times that would allow the entire HISs to converge to a single value. This work is presently ongoing.

## Figures and Tables

**Algorithm 1:**
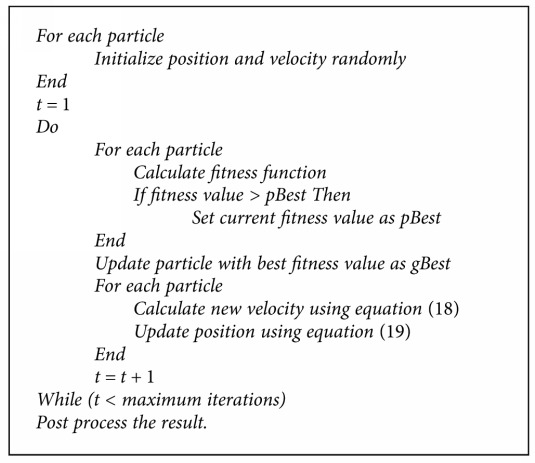
Pseudocode for particle swarm optimization.

**Algorithm 2:**
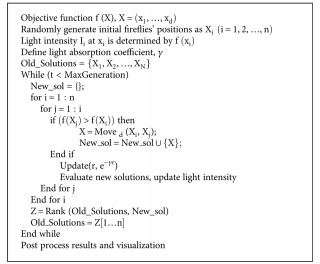
Pseudocode representation of the basic firefly algorithm.

**Algorithm 3:**
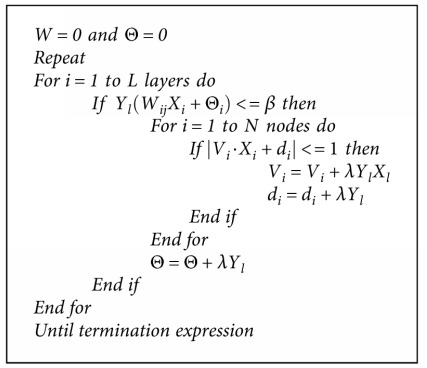
MLP training algorithm.

**Algorithm 4:**
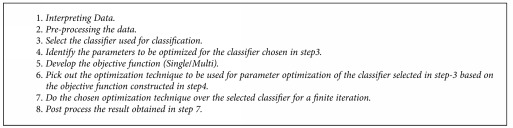
Basic steps of hybrid intelligent system.

**Table 1:** Comparison of basic, resampling, and ADABOOST versions of SVM and MLP.

Dataset	MLP	SVM	RMLP	RSVM	ADA-MLP	ADA-SVM
Cleveland	79.2079	82.8383	**93.72**	**85.0886**	76.23	82.5083
Statlog	77.4074	84.07	**86.6667**	**83.7037**	77.777	84.07
Spect	79.4	81.65	**88.38**	**88.764**	79.4007	80.8989
Spectf	76.03	79.40	**90.2622**	**82.397**	76.03	77.9026
Eric	77.99	78.95	**88.51**	**83.73**	**77.9904**	**77.9904**
WBC	95.28	96.85	**97.1388**	**96.5665**	95.5651	96.7096
Hepatitis	81.94	85.16	**90.3226**	**85.8065**	78.7097	78.9097
Thyroid	96.28	89.77	**98.1395**	**78.6047**	97.2093	**85.1163**
Parkinson	91.28	86.15	**96.4103**	**90.2564**	92.3077	87.6923
Pima Indian diabetics	75.13	77.47	**79.2969**	**76.0417**	73.9583	77.3438
BUPA liver	71.59	70.14	**68.1159**	**54.4928**	**71.3043**	62.029

R: filter-based supervised instance resampling; ADA: ADABOOST.

**Table 2:** Summary of datasets used.

S. number	Dataset	Size
1	Cleveland	303 × 14
2	Statlog	270 × 14
3	Spect	267 × 23
4	Spectf	267 × 45
5	Eric	209 × 8
6	WBC	699 × 10
7	Hepatitis	155 × 20
8	Thyroid	215 × 6
9	Parkinson	195 × 23
10	Pima Indian diabetics	768 × 9
11	BUPA	345 × 7

**Table 3:** Signed-rank test at LOS 0.01 on resampled data.

Dataset	Objectives	PSVM		GSVM		FSVM		PMLP		GMLP	
		*P* value	*H*	*P* value	*H*	*P* value	*H*	*P* value	*H*	*P* value	*H*
Cleveland	PAC	3.69*E*−05	1	3.69*E*−05	1	3.69*E*−05	1	8.75*E*−05	1	8.125*E*−5	1
	SEN	4.4*E*−05	1	2.98*E*−05	1	3.69*E*−05	1	6.875*E*−5	1	1.13*E*−5	1
	SPE	3.69*E*−05	1	4.4*E*−05	1	4.4*E*−05	1	8.125*E*−5	1	4.375*E*−5	1

Statlog	PAC	3.69*E*−05	1	2.98*E*−05	1	3.69*E*−05	1	6.11*E*−05	1	2.31*E*−05	1
	SEN	3.69*E*−05	1	3.69*E*−05	1	4.4*E*−05	1	3.69*E*−05	1	4.4*E*−05	1
	SPE	2.98*E*−05	1	4.4*E*−05	1	3.69*E*−05	1	4.4*E*−05	1	4.4*E*−05	1

Spect	PAC	4.4*E*−05	1	4.4*E*−05	1	4.4*E*−05	1	0.000963	1	5*E*−05	1
	SEN	4.4*E*−05	1	7.23*E*−05	1	4.4*E*−05	1	0.007533	1	0.009141	1
	SPE	2.98*E*−05	1	3.69*E*−05	1	3.69*E*−05	1	2.98*E*−05	1	3.69*E*−05	1

Spectf	PAC	2.98*E*−05	1	4.4*E*−05	1	3.69*E*−05	1	5.17*E*−05	1	0.000726	1
	SEN	4.4*E*−05	1	4.4*E*−05	1	8.51*E*−05	1	0.000629	1	0.00082	1
	SPE	4.4*E*−05	1	4.4*E*−05	1	3.69*E*−05	1	2.98*E*−05	1	0.000627	1

Eric	PAC	4.4*E*−05	1	4.4*E*−05	1	4.4*E*−05	1	0.875	0	3.69*E*−05	1
	SEN	4.4*E*−05	1	4.4*E*−05	1	4.4*E*−05	1	5.2*E*−05	1	3.69*E*−05	1
	SPE	3.69*E*−05	1	2.98*E*−05	1	2.98*E*−05	1	3.69*E*−05	1	3.69*E*−05	1

WBC	PAC	4.37*E*−05	1	3.55*E*−05	1	4.37*E*−05	1	1	0	5*E*−05	1
	SEN	4.4*E*−05	1	3.69*E*−05	1	4.4*E*−05	1	0.5625	0	0.006127	1
	SPE	4.4*E*−05	1	4.4*E*−05	1	4.4*E*−05	1	0.4375	0	6.11*E*−05	1

Hepatitis	PAC	4.4*E*−05	1	3.69*E*−05	1	3.69*E*−05	1	0.4375	0	8.4375	1
	SEN	4.37*E*−05	1	5.2*E*−05	1	3.69*E*−05	1	8.75*E*−6	1	5.7126*E*−5	1
	SPE	4.4*E*−05	1	3.69*E*−05	1	3.69*E*−05	1	1.15*E*−5	1	2.3123*E*−5	1

Thyroid	PAC	4.4*E*−05	1	3.69*E*−05	1	3.69*E*−05	1	4.4*E*−05	1	4.4*E*−05	1
	SEN	4.4*E*−05	1	4.4*E*−05	1	2.98*E*−05	1	4.4*E*−05	1	2.98*E*−05	1
	SPE	4.4*E*−05	1	3.69*E*−05	1	4.4*E*−05	1	6.577*E*−5	1	0.007533	1

Parkinson	PAC	4.4*E*−05	1	4.4*E*−05	1	3.69*E*−05	1	3.69*E*−05	1	4.37*E*−05	1
	SEN	4.4*E*−05	1	2.98*E*−05	1	4.4*E*−05	1	0.006855	1	3.2366*E*−5	1
	SPE	4.4*E*−05	1	4.4*E*−05	1	3.69*E*−05	1	2.98*E*−05	1	3.69*E*−05	1

Pima Indian diabetics	PAC	4.4*E*−05	1	4.4*E*−05	1	4.4*E*−05	1	3.69*E*−05	1	1*E*−04	1
	SEN	3.69*E*−05	1	3.69*E*−05	1	2.98*E*−05	1	3.69*E*−05	1	2.98*E*−05	1
	SPE	4.4*E*−05	1	4.4*E*−05	1	3.69*E*−05	1	3.69*E*−05	1	3.69*E*−05	1

BUPA liver disease	PAC	4.4*E*−05	1	3.69*E*−05	1	4.4*E*−05	1	4.4*E*−05	1	3.69*E*−05	1
	SEN	4.4*E*−05	1	3.69*E*−05	1	4.4*E*−05	1	4.4*E*−05	1	4.4*E*−05	1
	SPE	3.69*E*−05	1	3.69*E*−05	1	3.69*E*−05	1	2.31*E*−05	1	2.31*E*−05	1

**Table 4:** Signed-rank test at LOS 0.05 on resampled data.

Dataset	Objectives	PSVM		GSVM		FSVM		PMLP		GMLP	
		*P* value	*H*	*P* value	*H*	*P* value	*H*	*P* value	*H*	*P* value	*H*
Cleveland	PAC	3.69*E*−05	1	3.69*E*−05	1	3.69*E*−05	1	8.75*E*−5	1	8.125*E*−5	1
	SEN	4.4*E*−05	1	2.98*E*−05	1	3.69*E*−05	1	6.875*E*−5	1	1.542*E*−4	1
	SPE	3.69*E*−05	1	4.4*E*−05	1	4.4*E*−05	1	8.125*E*−5	1	4.375	1

Statlog	PAC	3.69*E*−05	1	2.98*E*−05	1	3.69*E*−05	1	6.11*E*−05	1	2.31*E*−05	1
	SEN	3.69*E*−05	1	3.69*E*−05	1	4.4*E*−05	1	3.69*E*−05	1	4.4*E*−05	1
	SPE	2.98*E*−05	1	4.4*E*−05	1	3.69*E*−05	1	4.4*E*−05	1	4.4*E*−05	1

Spect	PAC	4.4*E*−05	1	4.4*E*−05	1	4.4*E*−05	1	0.000963	1	5*E*−05	1
	SEN	4.4*E*−05	1	7.23*E*−05	1	4.4*E*−05	1	0.007533	1	0.009141	1
	SPE	2.98*E*−05	1	3.69*E*−05	1	3.69*E*−05	1	2.98*E*−05	1	3.69*E*−05	1

Spectf	PAC	2.98*E*−05	1	4.4*E*−05	1	3.69*E*−05	1	5.17*E*−05	1	0.000726	1
	SEN	4.4*E*−05	1	4.4*E*−05	1	8.51*E*−05	1	0.000629	1	0.00082	1
	SPE	4.4*E*−05	1	4.4*E*−05	1	3.69*E*−05	1	2.98*E*−05	1	0.000627	1

Eric	PAC	4.4*E*−05	1	4.4*E*−05	1	4.4*E*−05	1	8.75*E*−5	1	3.69*E*−05	1
	SEN	4.4*E*−05	1	4.4*E*−05	1	4.4*E*−05	1	5.2*E*−05	1	3.69*E*−05	1
	SPE	3.69*E*−05	1	2.98*E*−05	1	2.98*E*−05	1	3.69*E*−05	1	3.69*E*−05	1

WBC	PAC	4.37*E*−05	1	3.55*E*−05	1	4.37*E*−05	1	1.3982*E*−5	1	5.38*E*−05	1
	SEN	4.4*E*−05	1	3.69*E*−05	1	4.4*E*−05	1	5.625*E*−5	1	0.6127	0
	SPE	4.4*E*−05	1	4.4*E*−05	1	4.4*E*−05	1	4.235*E*−5	1	6.11*E*−05	1

Hepatitis	PAC	4.4*E*−05	1	3.69*E*−05	1	3.69*E*−05	1	4.375*E*−5	1	8.475*E*−5	1
	SEN	4.37*E*−05	1	5.2*E*−05	1	3.69*E*−05	1	0.875*E*−5	1	1.873*E*−5	1
	SPE	4.4*E*−05	1	3.69*E*−05	1	3.69*E*−05	1	1.321*E*−5	1	1.098*E*−5	1

Thyroid	PAC	4.4*E*−05	1	3.69*E*−05	1	3.69*E*−05	1	4.4*E*−05	1	4.4*E*−05	1
	SEN	4.4*E*−05	1	4.4*E*−05	1	2.98*E*−05	1	4.4*E*−05	1	2.98*E*−05	1
	SPE	4.4*E*−05	1	3.69*E*−05	1	4.4*E*−05	1	0.026577	1	0.007533	1

Parkinson	PAC	4.4*E*−05	1	4.4*E*−05	1	3.69*E*−05	1	3.69*E*−05	1	4.37*E*−05	1
	SEN	4.4*E*−05	1	2.98*E*−05	1	4.4*E*−05	1	0.006855	1	0.032366	1
	SPE	4.4*E*−05	1	4.4*E*−05	1	3.69*E*−05	1	2.98*E*−05	1	3.69*E*−05	1

Pima Indian diabetics	PAC	4.4*E*−05	1	4.4*E*−05	1	4.4*E*−05	1	3.69*E*−05	1	1*E*−04	1
	SEN	3.69*E*−05	1	3.69*E*−05	1	2.98*E*−05	1	3.69*E*−05	1	2.98*E*−05	1
	SPE	4.4*E*−05	1	4.4*E*−05	1	3.69*E*−05	1	3.69*E*−05	1	3.69*E*−05	1

BUPA liver disease	PAC	4.4*E*−05	1	3.69*E*−05	1	4.4*E*−05	1	4.4*E*−05	1	3.69*E*−05	1
	SEN	4.4*E*−05	1	3.69*E*−05	1	4.4*E*−05	1	4.4*E*−05	1	4.4*E*−05	1
	SPE	3.69*E*−05	1	3.69*E*−05	1	3.69*E*−05	1	2.31*E*−05	1	2.31*E*−05	1

**Table 5:** Signed-rank test at LOS 0.01 on without resampled data.

Dataset	Objectives	PSVM		GSVM		FSVM		PMLP		GMLP	
		*P* value	*H*	*P* value	*H*	*P* value	*H*	*P* value	*H*	*P* value	*H*
Cleveland	PAC	3.69*E*−05	1	3.69*E*−05	1	3.69*E*−05	1	2.98*E*−05	1	2.98*E*−05	1
	SEN	4.4*E*−05	1	2.98*E*−05	1	4.4*E*−05	1	0.004662	1	8.51*E*−05	1
	SPE	4.4*E*−05	1	4.4*E*−05	1	4.4*E*−05	1	4.4*E*−05	1	3.69*E*−05	1

Statlog	PAC	2.98*E*−05	1	3.69*E*−05	1	3.69*E*−05	1	3.69*E*−05	1	4.4*E*−05	1
	SEN	0.035645	0	3.69*E*−05	1	6.14*E*−05	1	4.4*E*−05	1	2.98*E*−05	1
	SPE	4.4*E*−05	1	4.4*E*−05	1	4.4*E*−05	1	4.4*E*−05	1	3.69*E*−05	1

Spect	PAC	3.69*E*−05	1	4.4*E*−05	1	4.4*E*−05	1	4.4*E*−05	1	3.69*E*−05	1
	SEN	8.5*E*−05	1	3.69*E*−05	1	3.69*E*−05	1	4.4*E*−05	1	5.17*E*−05	1
	SPE	4.4*E*−05	1	4.4*E*−05	1	3.69*E*−05	1	3.69*E*−05	1	3.69*E*−05	1

Spectf	PAC	4.4*E*−05	1	4.4*E*−05	1	3.69*E*−05	1	4.4*E*−05	1	3.69*E*−05	1
	SEN	2.98*E*−05	1	4.4*E*−05	1	4.4*E*−05	1	4.4*E*−05	1	4.4*E*−05	1
	SPE	4.4*E*−05	1	3.69*E*−05	1	4.4*E*−05	1	2.98*E*−05	1	3.69*E*−05	1

Eric	PAC	4.4*E*−05	1	4.4*E*−05	1	4.4*E*−05	1	3.69*E*−05	1	4.375*E*−05	1
	SEN	4.4*E*−05	1	4.4*E*−05	1	6.14*E*−05	1	0.000542	1	4.4*E*−05	1
	SPE	4.4*E*−05	1	3.69*E*−05	1	3.69*E*−05	1	4.37*E*−05	1	3.69*E*−05	1

WBC	PAC	7.23*E*−05	1	0.000117	1	0.000943	1	0.005267	1	1*E*−04	1
	SEN	3.69*E*−05	1	4.4*E*−05	1	3.69*E*−05	1	0.000544	1	8.51*E*−05	1
	SPE	0.007263	1	0.032366	0	0.000826	1	0.00003	1	4.37*E*−05	1

Hepatitis	PAC	4.4*E*−05	1	3.69*E*−05	1	4.4*E*−05	1	4.4*E*−05	1	4.4*E*−05	1
	SEN	3.69*E*−05	1	3.69*E*−05	1	3.69*E*−05	1	3.69*E*−05	1	3.69*E*−05	1
	SPE	5*E*−05	1	4.4*E*−05	1	6.11*E*−05	1	2.98*E*−05	1	3.69*E*−05	1

Thyroid	PAC	4.4*E*−05	1	4.4*E*−05	1	3.69*E*−05	1	4.4*E*−05	1	4.4*E*−05	1
	SEN	0.005738	1	0.000725	1	0.000726	1	3.69*E*−05	1	4.4*E*−05	1
	SPE	4.4*E*−05	1	4.4*E*−05	1	3.69*E*−05	1	4.37*E*−05	1	3.69*E*−05	1

Parkinson	PAC	4.4*E*−05	1	3.69*E*−05	1	4.4*E*−05	1	3.69*E*−05	1	4.4*E*−05	1
	SEN	3.69*E*−05	1	3.69*E*−05	1	3.69*E*−05	1	6.14*E*−05	1	4.359*E*−05	1
	SPE	4.4*E*−05	1	3.69*E*−05	1	4.4*E*−05	1	4.4*E*−05	1	3.69*E*−05	1

Pima Indian diabetics	PAC	4.4*E*−05	1	3.69*E*−05	1	3.69*E*−05	1	3.69*E*−05	1	4.4*E*−05	1
	SEN	3.69*E*−05	1	3.69*E*−05	1	3.69*E*−05	1	3.69*E*−05	1	3.69*E*−05	1
	SPE	3.69*E*−05	1	3.69*E*−05	1	3.69*E*−05	1	3.69*E*−05	1	3.55*E*−05	1

BUPA liver disease	PAC	4.4*E*−05	1	3.69*E*−05	1	4.4*E*−05	1	4.4*E*−05	1	3.69*E*−05	1
	SEN	4.4*E*−05	1	0.00509	1	0.00646	1	4.4*E*−05	1	4.4*E*−05	1
	SPE	3.69*E*−05	1	3.69*E*−05	1	3.69*E*−05	1	6.11*E*−05	1	3.69*E*−05	1

**Table 6:** Signed-rank test at LOS 0.05 on without resampled data.

Dataset	Objectives	PSVM		GSVM		FSVM		PMLP		GMLP	
		*P* value	*H*	*P* value	*H*	*P* value	*H*	*P* value	*H*	*P* value	*H*
Cleveland	PAC	3.69*E*−05	1	3.69*E*−05	1	3.69*E*−05	1	2.98*E*−05	1	2.98*E*−05	1
	SEN	4.4*E*−05	1	2.98*E*−05	1	4.4*E*−05	1	0.004662	1	8.51*E*−05	1
	SPE	4.4*E*−05	1	4.4*E*−05	1	4.4*E*−05	1	4.4*E*−05	1	3.69*E*−05	1

Statlog	PAC	2.98*E*−05	1	3.69*E*−05	1	3.69*E*−05	1	3.69*E*−05	1	4.4*E*−05	1
	SEN	0.035645	1	3.69*E*−05	1	6.14*E*−05	1	4.4*E*−05	1	2.98*E*−05	1
	SPE	4.4*E*−05	1	4.4*E*−05	1	4.4*E*−05	1	4.4*E*−05	1	3.69*E*−05	1

Spect	PAC	3.69*E*−05	1	4.4*E*−05	1	4.4*E*−05	1	4.4*E*−05	1	3.69*E*−05	1
	SEN	8.5*E*−05	1	3.69*E*−05	1	3.69*E*−05	1	4.4*E*−05	1	5.17*E*−05	1
	SPE	4.4*E*−05	1	4.4*E*−05	1	3.69*E*−05	1	3.69*E*−05	1	3.69*E*−05	1

Spectf	PAC	4.4*E*−05	1	4.4*E*−05	1	3.69*E*−05	1	4.4*E*−05	1	3.69*E*−05	1
	SEN	2.98*E*−05	1	4.4*E*−05	1	4.4*E*−05	1	4.4*E*−05	1	4.4*E*−05	1
	SPE	4.4*E*−05	1	3.69*E*−05	1	4.4*E*−05	1	2.98*E*−05	1	3.69*E*−05	1

Eric	PAC	4.4*E*−05	1	4.4*E*−05	1	4.4*E*−05	1	3.69*E*−05	1	0.4375	0
	SEN	4.4*E*−05	1	4.4*E*−05	1	6.14*E*−05	1	0.000542	1	4.4*E*−05	1
	SPE	4.4*E*−05	1	3.69*E*−05	1	3.69*E*−05	1	4.37*E*−05	1	3.69*E*−05	1

WBC	PAC	7.23*E*−05	1	0.000117	1	0.000943	1	0.005267	1	1*E*−04	1
	SEN	3.69*E*−05	1	4.4*E*−05	1	3.69*E*−05	1	0.000544	1	8.51*E*−05	1
	SPE	0.007263	1	0.032366	1	0.033826	1	0.039203	1	4.37*E*−05	1

Hepatitis	PAC	4.4*E*−05	1	3.69*E*−05	1	4.4*E*−05	1	4.4*E*−05	1	4.4*E*−05	1
	SEN	3.69*E*−05	1	3.69*E*−05	1	3.69*E*−05	1	3.69*E*−05	1	3.69*E*−05	1
	SPE	5*E*−05	1	4.4*E*−05	1	6.11*E*−05	1	2.98*E*−05	1	3.69*E*−05	1

Thyroid	PAC	4.4*E*−05	1	4.4*E*−05	1	3.69*E*−05	1	4.4*E*−05	1	4.4*E*−05	1
	SEN	0.005738	1	0.000725	1	0.000726	1	3.69*E*−05	1	4.4*E*−05	1
	SPE	4.4*E*−05	1	4.4*E*−05	1	3.69*E*−05	1	4.37*E*−05	1	3.69*E*−05	1

Parkinson	PAC	4.4*E*−05	1	3.69*E*−05	1	4.4*E*−05	1	3.69*E*−05	1	4.4*E*−05	1
	SEN	3.69*E*−05	1	3.69*E*−05	1	3.69*E*−05	1	6.14*E*−05	1	0.043059	1
	SPE	4.4*E*−05	1	3.69*E*−05	1	4.4*E*−05	1	4.4*E*−05	1	3.69*E*−05	1

Pima Indian diabetics	PAC	4.4*E*−05	1	3.69*E*−05	1	3.69*E*−05	1	3.69*E*−05	1	4.4*E*−05	1
	SEN	3.69*E*−05	1	3.69*E*−05	1	3.69*E*−05	1	3.69*E*−05	1	3.69*E*−05	1
	SPE	3.69*E*−05	1	3.69*E*−05	1	3.69*E*−05	1	3.69*E*−05	1	3.55*E*−05	1

BUPA liver disease	PAC	4.4*E*−05	1	3.69*E*−05	1	4.4*E*−05	1	4.4*E*−05	1	3.69*E*−05	1
	SEN	4.4*E*−05	1	0.00509	1	0.00646	1	4.4*E*−05	1	4.4*E*−05	1
	SPE	3.69*E*−05	1	3.69*E*−05	1	3.69*E*−05	1	6.11*E*−05	1	3.69*E*−05	1

**Table 7:** Results of Student's *t*-test on without resampled data.

Dataset name	Obj	Alpha = 0.01				Alpha = 0.05			
		*H*	*P* value	Lower bound	Upper bound	*H*	*P* value	Lower bound	Upper bound
Cleveland	PAC	0	0.107409014	93.63760552	94.15613048	0	0.107409014	93.70719481	94.08654119
	SEN	0	0.119440215	93.37174786	93.52239194	0	0.119440215	93.39196524	93.50217457
	SPE	0	0.110406058	94.4269811	94.85972715	0	0.110406058	94.48505832	94.80164993

Statlog	PAC	0	0.198452296	89.40087856	89.71350963	0	0.198452296	89.4428356	89.67155259
	SEN	0	0.113684201	87.8202939	88.13645358	0	0.113684201	87.8627245	88.09402298
	SPE	0	0.085172965	90.21689475	90.78345951	0	0.085172965	90.29293128	90.70742299

Spect	PAC	0	0.09899382	88.79823642	89.22375777	0	0.09899382	88.85534404	89.16665015
	SEN	0	0.102037465	91.52271275	91.98167028	0	0.102037465	91.58430772	91.92007531
	SPE	0	0.094031375	75.19513272	75.64666638	0	0.094031375	75.25573136	75.58606775

Spectf	PAC	0	0.091447714	89.90632221	90.34258368	0	0.091447714	89.96487122	90.28403467
	SEN	0	0.085170746	93.2108793	93.62575965	0	0.085170746	93.26655883	93.57008011
	SPE	0	0.086020283	75.50805229	76.1105871	0	0.086020283	75.58891622	76.02972317

Eric	PAC	0	0.123534674	89.66246147	90.03031881	0	0.123534674	89.71183022	89.98095006
	SEN	0	0.127198304	87.50689214	87.66497294	0	0.127198304	87.52810757	87.64375751
	SPE	0	0.166768736	91.88301746	91.98548668	0	0.166768736	91.89676947	91.97173467

WBC	PAC	0	0.104809721	97.89345297	98.02702302	0	0.104809721	97.91137891	98.00909708
	SEN	0	0.103386039	96.30307823	96.68720924	0	0.103386039	96.35463101	96.63565646
	SPE	0	0.108706462	98.37053699	98.78539395	0	0.108706462	98.42621338	98.72971756

Hepatitis	PAC	0	0.131198082	92.0423242	92.32294757	0	0.131198082	92.07998561	92.28528616
	SEN	0	0.270421552	80.5671699	80.85759447	0	0.270421552	80.60614669	80.81861768
	SPE	0	0.141041289	94.26694606	94.66132599	0	0.141041289	94.31987431	94.60839774

Thyroid	PAC	0	0.171022773	97.15734101	97.84211907	0	0.171022773	97.2492425	97.75021758
	SEN	0	0.189226475	94.54448673	94.93137205	0	0.189226475	94.59640916	94.87944963
	SPE	0	0.209391475	98.4352079	98.84065802	0	0.209391475	98.48962183	98.78624408

Parkinson	PAC	0	0.100403912	96.04769583	96.49955298	0	0.100403912	96.10833788	96.43891093
	SEN	0	0.118064733	97.14392547	97.46967293	0	0.118064733	97.18764281	97.42595559
	SPE	0	0.132191436	92.30860837	92.78786405	0	0.132191436	92.37292747	92.72354494

Pima Indian diabetics	PAC	0	0.092109816	80.171399	80.70052364	0	0.092109816	80.24241083	80.62951182
	SEN	0	0.085031456	76.12631776	76.69348033	0	0.085031456	76.20243452	76.61736358
	SPE	0	0.165042763	81.90646042	82.11053899	0	0.165042763	81.93384904	82.08315037

BUPA liver disease	PAC	0	0.155271161	70.23491732	70.49206105	0	0.155271161	70.26942761	70.45755076
	SEN	0	0.114021708	67.20708253	67.52742545	0	0.114021708	67.25007455	67.48443343
	SPE	0	0.145476728	73.12029125	73.35511283	0	0.145476728	73.15180577	73.32359831

**Table 8:** Results of Student's *t*-test on resampled data.

Dataset name	Obj	Alpha = 0.01				Alpha = 0.05			
		*H*	*P* value	Lower bound	Upper bound	*H*	*P* value	Lower bound	Upper bound
Cleveland	PAC	0	0.107409014	93.63760552	94.15613048	0	0.107409014	93.70719481	94.08654119
	SEN	0	0.119440215	93.37174786	93.52239194	0	0.119440215	93.39196524	93.50217457
	SPE	0	0.110406058	94.4269811	94.85972715	0	0.110406058	94.48505832	94.80164993

Statlog	PAC	0	0.198452296	89.40087856	89.71350963	0	0.198452296	89.4428356	89.67155259
	SEN	0	0.113684201	87.8202939	88.13645358	0	0.113684201	87.8627245	88.09402298
	SPE	0	0.085172965	90.21689475	90.78345951	0	0.085172965	90.29293128	90.70742299

Spect	PAC	0	0.09899382	88.79823642	89.22375777	0	0.09899382	88.85534404	89.16665015
	SEN	0	0.102037465	91.52271275	91.98167028	0	0.102037465	91.58430772	91.92007531
	SPE	0	0.094031375	75.19513272	75.64666638	0	0.094031375	75.25573136	75.58606775

Spectf	PAC	0	0.091447714	89.90632221	90.34258368	0	0.091447714	89.96487122	90.28403467
	SEN	0	0.085170746	93.2108793	93.62575965	0	0.085170746	93.26655883	93.57008011
	SPE	0	0.086020283	75.50805229	76.1105871	0	0.086020283	75.58891622	76.02972317

Eric	PAC	0	0.123534674	89.66246147	90.03031881	0	0.123534674	89.71183022	89.98095006
	SEN	0	0.127198304	87.50689214	87.66497294	0	0.127198304	87.52810757	87.64375751
	SPE	0	0.166768736	91.88301746	91.98548668	0	0.166768736	91.89676947	91.97173467

WBC	PAC	0	0.104809721	97.89345297	98.02702302	0	0.104809721	97.91137891	98.00909708
	SEN	0	0.103386039	96.30307823	96.68720924	0	0.103386039	96.35463101	96.63565646
	SPE	0	0.108706462	98.37053699	98.78539395	0	0.108706462	98.42621338	98.72971756

Hepatitis	PAC	0	0.131198082	92.0423242	92.32294757	0	0.131198082	92.07998561	92.28528616
	SEN	0	0.270421552	80.5671699	80.85759447	0	0.270421552	80.60614669	80.81861768
	SPE	0	0.141041289	94.26694606	94.66132599	0	0.141041289	94.31987431	94.60839774

Thyroid	PAC	0	0.171022773	97.15734101	97.84211907	0	0.171022773	97.2492425	97.75021758
	SEN	0	0.189226475	94.54448673	94.93137205	0	0.189226475	94.59640916	94.87944963
	SPE	0	0.209391475	98.4352079	98.84065802	0	0.209391475	98.48962183	98.78624408

Parkinson	PAC	0	0.100403912	96.04769583	96.49955298	0	0.100403912	96.10833788	96.43891093
	SEN	0	0.118064733	97.14392547	97.46967293	0	0.118064733	97.18764281	97.42595559
	SPE	0	0.132191436	92.30860837	92.78786405	0	0.132191436	92.37292747	92.72354494

Pima Indian diabetics	PAC	0	0.092109816	80.171399	80.70052364	0	0.092109816	80.24241083	80.62951182
	SEN	0	0.085031456	76.12631776	76.69348033	0	0.085031456	76.20243452	76.61736358
	SPE	0	0.165042763	81.90646042	82.11053899	0	0.165042763	81.93384904	82.08315037

BUPA liver disease	PAC	0	0.155271161	70.23491732	70.49206105	0	0.155271161	70.26942761	70.45755076
	SEN	0	0.114021708	67.20708253	67.52742545	0	0.114021708	67.25007455	67.48443343
	SPE	0	0.145476728	73.12029125	73.35511283	0	0.145476728	73.15180577	73.32359831

**Table 9:** Performance metric values on all datasets with and without resampling.

Dataset name	Without resampling		Resampling		Dataset name	Without resampling		Resampling	
	GD	SP	GD	SP		GD	SP	GD	SP
Cleveland	0.21	0.8	0.19	0.9	WBC	0.39	1.25	0.25	2.21
Statlog	0.39	1.02	0.16	1.91	Hepatitis	0.24	3.21	0.27	1.29
Spect	0.11	1.87	0.14	1.23	Thyroid	0.1	1.34	0.26	1.76
Spectf	0.18	1.98	0.12	1.2	Parkinson	0.22	1.8	0.18	2.92
Eric	0.29	2.02	0.13	1.92	Pima Indian diabetics	0.2	0.98	0.13	1.05
					BUPA	0.18	2.2	0.14	1.9

**Table 10:** Performance of hybrid systems on Cleveland dataset.

Cleveland								
	Resampling	Without resampling	Resampling			Without resampling		
			PSO	GSA	FA	PSO	GSA	FA
	Basic SVM		Parameter optimized SVM					
PAC	85.14	82.83	86.79	86.13	86.13	83.49	82.83	83.49
Sensitivity	83.33	82.18	84.91	84.74	84.35	82.38	82.18	82.38
Specificity	87.80	83.72	89.51	88.09	88.70	85.03	83.72	85.03
*F*-measure	85.04	82.77	86.71	86.06	86.05	83.41	82.77	83.41
Recall	85.14	82.83	86.79	86.13	86.13	83.49	82.83	83.49
Precision	85.36	82.8	87.01	86.27	86.33	83.60	82.88	83.60
	Basic MLP		Parameter optimized MLP					
PAC	93.72	79.20	**94.05**	**94.05**	**94.05**	85.14	84.15	**85.80**
Sensitivity	93.45	80.23	**93.49**	**93.49**	**93.49**	**84.79**	84.11	84.57
Specificity	94.07	77.94	**94.77**	**94.77**	**94.77**	85.60	84.21	**87.5**
*F*-measure	93.72	79.18	**94.05**	**94.05**	**94.05**	85.11	84.12	**85.74**
Recall	93.72	79.20	**94.05**	**94.05**	**94.05**	85.14	84.15	**85.80**
Precision	93.73	79.18	**94.07**	**94.07**	**94.07**	85.16	84.16	**85.91**

**Table 11:** Comparison of hybrid systems with HMV [14] for Cleveland dataset.

	Method	Accuracy	Sensitivity	Specificity	*F*-measure
Base paper	Ensemble	85	83.82	88.41	82.15
Without resampling	FMLP	85.8	87.5	84.6	87.5
Resampling	FMLP (PMLP)	94.1	94.8	93.5	94.1

**Table 12:** Performance of hybrid systems on Statlog dataset.

Statlog								
	Resampling	Without resampling	Resampling			Without resampling		
			PSO	GSA	FA	PSO	GSA	FA
	Basic SVM		Parameter optimized SVM					
PAC	85.19	84.44	85.56	85.56	85.56	84.44	84.81	84.44
Sensitivity	83.81	83.62	82.73	83.96	83.96	83.62	84.35	84.21
Specificity	86.06	85.06	87.50	86.59	86.59	85.06	85.16	84.62
*F*-measure	85.12	84.41	85.55	85.50	85.50	84.41	84.78	84.40
Recall	85.19	84.44	85.56	85.56	85.56	84.44	84.81	84.44
Precision	85.14	84.42	85.54	85.51	85.51	84.42	84.80	84.44
	Basic MLP		Parameter optimized MLP					
PAC	86.67	77.41	90.37	**90.74**	89.63	84.07	81.85	**85.93**
Sensitivity	82.61	73.60	91.26	**92.16**	88.07	**85.32**	79.34	83.61
Specificity	89.68	80.69	89.82	89.88	**90.68**	83.23	83.89	**87.84**
*F*-measure	86.70	77.45	90.31	**90.67**	89.61	83.97	81.86	**85.94**
Recall	86.67	77.41	90.37	**90.74**	89.63	84.07	81.85	**85.93**
Precision	86.77	77.54	90.41	**90.82**	89.61	84.16	81.87	**85.96**

**Table 13:** Comparison of hybrid systems with BagMOOV [15] for Statlog dataset.

	Method	Accuracy	Sensitivity	Specificity	*F*-measure
Base paper	Ensemble	84.4	86	86	86
Without resampling	FMLP	85.9	83.6	87.8	85.9
Resampling	GMLP	90.74	92.2	89.9	90.7

**Table 14:** Performance of hybrid systems on Spect dataset.

Spect								
	Resampling	Without resampling	Resampling			Without resampling		
			PSO	GSA	FA	PSO	GSA	FA
	Basic SVM		Parameter optimized SVM					
PAC	88.39	81.65	88.39	88.39	88.39	82.77	82.40	82.77
Sensitivity	91.07	88.26	91.07	91.44	91.07	87.05	88.02	**88.43**
Specificity	74.42	55.56	74.42	73.33	74.42	60.47	58.00	58.82
*F*-measure	87.96	81.59	87.96	88.06	87.96	81.95	82.08	82.53
Recall	88.39	81.65	88.39	88.39	88.39	82.77	82.40	82.77
Precision	87.83	81.53	87.83	87.91	87.83	81.58	81.83	82.33
	Basic MLP		Parameter optimized MLP					
PAC	88.39	79.40	89.51	**89.51**	89.14	82.40	83.52	**85.02**
Sensitivity	91.44	87.56	91.93	**91.93**	91.89	87.33	87.17	86.44
Specificity	73.33	50.00	**77.27**	**77.27**	75.56	58.70	63.41	**74.19**
*F*-measure	88.06	79.60	89.17	**89.17**	88.83	81.80	82.58	**83.33**
Recall	88.39	79.40	89.51	**89.51**	89.14	82.40	83.52	**85.02**
Precision	87.91	79.82	89.07	**89.07**	88.71	81.43	82.28	**83.92**

**Table 15:** Comparison of hybrid systems with BagMOOV [15] for Spect dataset.

	Method	Accuracy	Sensitivity	Specificity	*F*-measure
Base paper	Ensemble	82.02	27.27	96.23	42.50
Without resampling	FMLP	85	86.4	74.2	83.3
Resampling	FMLP	89.5	91.9	77.3	89.2

**Table 16:** Performance of hybrid systems on Spectf dataset.

Spectf								
	Resampling	Without resampling	Resampling			Without resampling		
			PSO	GSA	FA	PSO	GSA	FA
	Basic SVM		Parameter optimized SVM					
PAC	87.64	79.40	89.14	89.14	88.01	80.90	79.40	80.15
Sensitivity	92.92	87.56	94.71	**94.71**	92.96	87.10	87.56	**88.04**
Specificity	67.27	50.00	69.49	69.49	68.52	54.00	50.00	51.72
*F*-measure	87.77	79.60	89.39	89.39	88.10	**80.56**	79.60	80.34
Recall	87.64	79.40	89.14	89.14	88.01	80.90	79.40	80.15
Precision	87.93	79.82	89.80	89.80	88.20	80.28	79.82	80.56
	Basic MLP		Parameter optimized MLP					
PAC	90.26	76.03	**90.64**	89.89	90.26	80.52	80.90	**82.40**
Sensitivity	93.15	85.24	93.58	93.12	93.55	87.74	84.85	82.35
Specificity	77.08	42.11	**77.55**	75.51	76.00	52.73	55.56	**83.33**
*F*-measure	90.11	76.19	**90.53**	89.77	90.19	80.52	79.31	77.56
Recall	90.26	76.03	**90.64**	89.89	90.26	80.52	80.90	**82.40**
Precision	90.02	76.35	**90.46**	89.69	90.13	80.52	78.81	**82.55**

**Table 17:** Comparison of hybrid systems with BagMOOV [15] for Spectf dataset.

	Method	Accuracy	Sensitivity	Specificity	*F*-measure
Base paper	Ensemble	78.28	7.27	96.70	13.53
Without resampling	FMLP	82.4	82.4	83.3	77.6
Resampling	PMLP	90.6	93.6	77.6	90.5

**Table 18:** Performance of hybrid systems on Eric dataset.

Eric								
	Resampling	Without resampling	Resampling			Without resampling		
			PSO	GSA	FA	PSO	GSA	FA
	Basic SVM		Parameter optimized SVM					
PAC	84.21	78.95	85.17	85.65	85.17	80.86	78.95	80.86
Sensitivity	80.20	83.33	80.00	81.37	81.19	86.11	83.33	86.11
Specificity	87.96	76.64	90.38	89.72	88.89	78.10	76.64	78.10
*F*-measure	84.25	78.49	85.20	85.68	85.20	80.45	78.49	80.45
Recall	84.21	78.95	85.17	85.65	85.17	80.86	78.95	80.86
Precision	84.47	79.59	85.71	85.97	85.43	81.63	79.59	81.63
	Basic MLP		Parameter optimized MLP					
PAC	88.52	77.99	89.95	**91.39**	89.95	80.86	81.34	**81.34**
Sensitivity	85.00	77.38	86.87	**88.78**	87.63	85.14	**88.41**	85.33
Specificity	91.74	78.40	92.73	**93.69**	91.96	78.52	77.86	**79.10**
*F*-measure	88.54	77.85	89.97	**91.40**	89.96	80.51	80.84	**81.02**
Recall	88.52	77.99	89.95	**91.39**	89.95	80.86	81.34	**81.34**
Precision	88.71	77.95	90.09	**91.48**	90.01	81.43	**82.50**	81.85

**Table 19:** Comparison of hybrid systems with BagMOOV [15] for Eric dataset.

	Method	Accuracy	Sensitivity	Specificity	*F*-measure
Base paper	Ensemble	80.86	86.32	73.91	79.64
Without resampling	FMLP	81.3	88.4	77.9	80.8
Resampling	GMLP	91.4	88.8	93.7	91.4

**Table 20:** Performance of hybrid systems on WBC dataset.

Breast cancer								
	Resampling	Without resampling	Resampling			Without resampling		
			PSO	GSA	FA	PSO	GSA	FA
	Basic SVM		Parameter optimized SVM					
PAC	96.14	96.85	97.00	97.00	97.00	**97.00**	96.85	96.85
Sensitivity	92.59	94.69	92.43	92.43	92.43	**95.08**	94.69	94.69
Specificity	98.03	98.02	**99.55**	**99.55**	**99.55**	**98.02**	98.02	98.02
*F*-measure	96.15	96.86	97.02	97.02	97.02	**97.00**	96.86	96.86
Recall	96.14	96.85	97.00	97.00	97.00	**97.00**	96.85	96.85
Precision	96.21	96.87	97.17	97.17	97.17	97.01	96.87	96.87
	Basic MLP		Parameter optimized MLP					
PAC	97.14	95.28	98.00	97.71	**98.00**	96.57	**97.00**	96.42
Sensitivity	95.34	92.62	**96.61**	96.58	**96.61**	93.93	94.35	93.55
Specificity	98.06	96.70	98.70	98.28	98.70	98.01	98.45	98.00
*F*-measure	97.14	95.29	**98.00**	97.71	**98.00**	96.58	97.01	96.44
Recall	97.14	95.28	**98.00**	97.71	**98.00**	96.57	**97.00**	96.42
Precision	97.15	95.30	**98.00**	97.71	**98.00**	96.60	**97.04**	96.47

**Table 21:** Comparison of hybrid systems with HMV [14] for WBC dataset.

	Method	Accuracy	Sensitivity	Specificity	*F*-measure
Base paper	Ensemble	96.71	98.01	96.94	97.48
Without resampling	PSVM	97	95.1	98	97
Resampling	PSVM	98	96.6	98.7	98

**Table 22:** Performance of hybrid systems on hepatitis dataset.

Hepatitis								
	Resampling	Without resampling	Resampling			Without resampling		
			PSO	GSA	FA	PSO	GSA	FA
	Basic SVM		Parameter optimized SVM					
PAC	89.03	85.16	89.68	89.68	89.68	**87.10**	**87.10**	**87.10**
Sensitivity	73.91	65.52	80.00	80.00	80.00	**73.08**	**73.08**	**73.08**
Specificity	91.67	89.68	91.11	91.11	91.11	89.92	89.92	89.92
*F*-measure	88.60	84.89	88.97	88.97	88.97	86.58	86.58	86.58
Recall	89.03	85.16	89.68	89.68	89.68	87.10	87.10	87.10
Precision	88.46	84.69	89.10	89.10	89.10	86.44	86.44	86.44
	Basic MLP		Parameter optimized MLP					
PAC	90.32	81.94	**92.26**	**92.26**	**92.26**	87.10	85.16	83.23
Sensitivity	72.41	56.67	**80.77**	**80.77**	**80.77**	71.43	71.43	59.38
Specificity	94.44	88.00	**94.57**	**94.57**	**94.57**	**90.55**	87.31	89.43
*F*-measure	90.39	81.72	**92.14**	**92.14**	**92.14**	**86.77**	83.94	83.23
Recall	90.32	81.94	**92.26**	**92.26**	**92.26**	**87.10**	85.16	83.23
Precision	90.46	81.53	**92.08**	**92.08**	**92.08**	**86.60**	84.03	83.23

**Table 23:** Comparison of hybrid systems with HMV [14] for hepatitis dataset.

	Method	Accuracy	Specificity	Sensitivity	*F*-measure
Base paper	Ensemble	86.45	90.48	92.68	91.57
Without resampling	PMLP	87.1	90.6	71.4	86.8
Resampling	PMLP	92.3	80.8	94.6	92.1

**Table 24:** Performance of hybrid systems on thyroid dataset.

Thyroid								
	Resampling	Without resampling	Resampling			Without resampling		
			PSO	GSA	FA	PSO	GSA	FA
	Basic SVM		Parameter optimized SVM					
PAC	89.77	89.77	91.16	91.16	91.16	89.77	89.77	89.77
Sensitivity	90.70	93.88	97.50	97.50	97.50	93.88	93.88	93.88
Specificity	89.53	88.55	89.71	89.71	89.71	88.55	88.55	88.55
*F*-measure	89.27	89.31	90.61	90.61	90.61	89.31	89.31	89.31
Recall	89.77	89.77	91.16	91.16	91.16	89.77	89.77	89.77
Precision	89.84	90.16	91.78	91.78	91.78	90.16	90.16	90.16
	Basic MLP		Parameter optimized MLP					
PAC	98.14	96.28	**98.60**	97.67	**98.6**	97.67	96.74	**97.67**
Sensitivity	98.18	93.85	**98.21**	94.83	**98.21**	**95.45**	93.94	**95.45**
Specificity	98.13	97.33	**98.74**	98.73	**98.74**	98.66	97.99	**98.66**
*F*-measure	98.13	96.28	**98.60**	97.68	**98.6**	**97.68**	96.75	**97.68**
Recall	98.14	96.28	**98.60**	97.67	**98.6**	**97.67**	96.74	**97.67**
Precision	98.14	96.28	**98.60**	97.69	**98.6**	**97.69**	96.76	**97.69**

**Table 25:** Comparison of hybrid systems with neural network [16] for thyroid dataset.

	Method	Accuracy	Sensitivity	Specificity	*F*-measure
Base paper	Neural networks	94.81	NIL	NIL	NIL
Without resampling	FMLP	97.7	95.5	98.7	97.7
Resampling	FMLP	98.6	98.2	98.7	98.6

**Table 26:** Performance of hybrid systems on Parkinson dataset.

Parkinson's disease								
	Resampling	Without resampling	Resampling			Without resampling		
			PSO	GSA	FA	PSO	GSA	FA
	Basic SVM		Parameter optimized SVM					
PAC	91.28	86.15	91.28	91.28	91.28	87.69	87.69	87.69
Sensitivity	90.00	87.04	90.00	90.00	90.00	86.39	86.39	86.39
Specificity	100.00	81.82	**100.00**	**100.00**	**100.00**	**96.15**	**96.15**	**96.15**
*F*-measure	90.41	85.21	90.41	90.41	90.41	86.29	86.29	86.29
Recall	91.28	86.15	91.28	91.28	91.28	87.69	87.69	87.69
Precision	92.15	85.75	92.15	92.15	92.15	88.79	88.79	88.79
	Basic MLP		Parameter optimized MLP					
PAC	96.41	91.28	96.92	**96.92**	96.41	92.31	92.31	**93.85**
Sensitivity	97.40	94.52	**97.42**	**97.42**	**97.40**	95.83	96.48	**96.55**
Specificity	92.68	81.63	95.00	95.00	92.68	82.35	81.13	86.00
*F*-measure	96.39	91.31	**96.90**	**96.90**	96.39	92.38	92.43	**93.89**
Recall	96.41	91.28	**96.92**	**96.92**	96.41	92.31	92.31	**93.85**
Precision	96.39	91.35	**96.90**	**96.90**	96.39	92.52	92.70	**93.95**

**Table 27:** Comparison of hybrid systems with HMV [14] for Parkinson dataset.

	Method	Accuracy	Sensitivity	Specificity	*F*-measure
Base paper	Ensemble	89.23	91.45	94.56	92.98
Without resampling	FMLP	93.8	96.6	86	93.9
Resampling	GMLP	96.9	97.4	95	96.9

**Table 28:** Performance of hybrid systems on Pima Indian diabetics' dataset.

Pima Indian diabetics								
	Resampling	Without resampling	Resampling			Without resampling		
			PSO	GSA	FA	PSO	GSA	FA
	Basic SVM		Parameter optimized SVM					
PAC	77.08	77.47	77.08	77.21	77.60	**78.26**	**78.26**	78.13
Sensitivity	72.78	72.51	72.53	72.93	73.63	74.63	74.63	**74.51**
Specificity	78.40	79.35	78.50	78.53	78.84	79.57	79.57	79.43
*F*-measure	75.86	76.74	75.90	76.01	76.45	**77.45**	**77.45**	77.30
Recall	77.08	77.47	77.08	77.21	77.60	**78.26**	**78.26**	78.13
Precision	76.51	76.97	76.49	76.65	77.09	**77.85**	**77.85**	77.71
	Basic MLP		Parameter optimized MLP					
PAC	79.30	75.13	77.47	80.60	**80.99**	74.48	77.60	76.04
Sensitivity	70.71	65.34	69.41	**76.59**	73.14	64.06	70.87	65.79
Specificity	83.18	79.88	80.69	82.06	**84.60**	79.69	80.48	**81.47**
*F*-measure	79.09	74.93	76.97	79.97	**80.83**	74.34	77.16	76.02
Recall	79.30	75.13	77.47	80.60	**80.99**	74.48	77.60	76.04
Precision	78.99	74.81	76.90	80.22	**80.75**	74.24	77.13	76.00

**Table 29:** Comparison of hybrid systems with HMV [14] for Pima Indian diabetics' dataset.

	Method	Accuracy	Sensitivity	Specificity	*F*-measure
Base paper	Ensemble	77.08	78.93	88.4	83.4
Without resampling	PSVM	78.3	74.6	79.6	77.5
Resampling	FMLP	81	73.1	84.6	80.8

**Table 30:** Performance of hybrid systems on BUPA liver disease dataset.

BUPA liver disease								
	Resampling	Without resampling	Resampling			Without resampling		
			PSO	GSA	FA	PSO	GSA	FA
	Basic SVM		Parameter optimized SVM					
PAC	63.48	70.14	66.09	66.67	63.48	70.72	70.14	70.14
Sensitivity	61.44	68.10	64.86	65.97	61.44	**72.00**	68.10	68.10
Specificity	65.10	71.18	67.01	67.16	65.10	70.20	71.18	71.18
*F*-measure	63.40	69.50	65.95	66.47	63.40	69.52	69.50	69.50
Recall	63.48	70.14	66.09	66.67	63.48	70.72	70.14	70.14
Precision	63.39	69.89	66.01	66.61	63.39	70.96	69.89	69.89
	Basic MLP		Parameter optimized MLP					
PAC	68.12	71.59	65.80	**73.33**	70.43	72.75	**73.04**	71.59
Sensitivity	63.93	69.75	61.88	68.85	**67.46**	70.73	70.00	68.50
Specificity	72.84	72.57	70.12	**78.40**	73.30	73.87	**74.88**	73.39
*F*-measure	68.12	71.06	65.81	**73.34**	70.46	72.35	**72.80**	71.27
Recall	68.12	71.59	65.80	**73.33**	70.43	72.75	**73.04**	71.59
Precision	68.68	71.38	66.27	**73.94**	70.57	72.55	**72.83**	71.34

**Table 31:** Comparison of hybrid systems with HMV [14] for BUPA liver disease dataset.

	Method	Accuracy	Sensitivity	Specificity	*F*-measure
Base paper	Ensemble	67.54	68.54	42.07	52.14
Without resampling	GMLP	73	70	74.9	72.8
Resampling	GMLP	73.3	68.9	78.4	73.3

**Table 32:** Sensitivity improvement with hybrid systems.

S. number	Set	Sensitivity				
		Base paper	Without resampling		Resampling	
			Technique	Percentage	Technique	Percentage
1	Cleveland	83.82	FMLP	**87.5**	FMLP	**94.8**
2	Statlog	86	PMLP	85.3	GMLP	**92.2**
3	Spect	27.27	FSVM	**88.4**	FMLP	**91.9**
4	Spectf	7.27	FSVM	**88**	GSVM	**94.7**
5	Eric	86.32	GMLP	**88.4**	GMLP	**88.8**
6	WBC	98.01	PSVM	95.1	FMLP	96.6
7	Hepatitis	90.48	FSVM	73.1	FMLP	80.8
8	Thyroid	**NIL**	GMLP	**95.5**	GMLP	**98.2**
9	Parkinson	91.45	FMLP	**96.6**	FMLP	**97.4**
10	Pima Indian diabetics	78.93	PSVM	74.6	FMLP	76.6
11	BUPA	68.54	PSVM	**72**	FMLP	67.5

**Table 33:** Specificity improvement with hybrid systems.

S. number	Dataset	Specificity				
		Base paper	Without resampling		Resampling	
			Technique	Percentage	Technique	Percentage
1	Cleveland	88.41	PMLP	84.8	FMLP	**93.5**
2	Statlog	86	FMLP	**87.8**	FMLP	**90.7**
3	Spect	96.23	FMLP	74.2	GMLP	77.3
4	Spectf	96.7	FMLP	83.3	PMLP	77.6
5	Eric	73.91	FMLP	**79.1**	GMLP	**93.7**
6	WBC	96.94	FMLP	**98**	FMLP	**98.7**
7	Hepatitis	92.68	PMLP	90.6	FMLP	**94.6**
8	Thyroid	**NIL**	GMLP	**98.7**	FMLP	**98.7**
9	Parkinson	94.56	FSVM	**96.2**	FSVM	**100**
10	Pima Indian diabetics	88.4	FMLP	81.5	GMLP	84.6
11	BUPA	42.07	GMLP	**74.9**	GMLP	**78.4**

**Table 34:** Parameters to be used in MLP for all datasets.

Dataset	Learning rate	Momentum	Hybrid MLP accuracy	Base paper accuracy
Cleveland	0.4410246832765716	0.945131728055943	85.8	85
Statlog	0.24687115044065697	0.7512112614957723	85.9	84.4
Spect	0.001620407197768992	0.5458467309532906	85	82.02
Spectf	0.0037254964036241137	0.6064034495456784	82.4	78.28
Eric	0.6953073769599724	0.9167657941184544	81.3	80.86
Breast cancer	0.5272364248697747	0.9288899224295802	96.99	96.71
Hepatitis	0.6376255545427609	0.9250563419048221	87.1	86.45
Thyroid	0.1516498076389815	0.48805304429332785	97.7	94.81
Parkinson	0.8486064853474067	0.3499016503919223	93.8	89.23
Pima Indian diabetics	0.03218577681226653	0.06466339445401592	77.60	77.08
BUPA	0.8329619224653821	0.014749643317800043	73	67.54
